# Identification of a bile acid and bile salt metabolism-related lncRNA signature for predicting prognosis and treatment response in hepatocellular carcinoma

**DOI:** 10.1038/s41598-023-46805-6

**Published:** 2023-11-09

**Authors:** Hao Cui, Jia Lian, Baiguo Xu, Zhenjun Yu, Huiling Xiang, Jingxiang Shi, Yingtang Gao, Tao Han

**Affiliations:** 1 Department of Gastroenterology and Hepatology, The Third Central Clinical College of Tianjin Medical University, Tianjin, China; 2 Department of Gastroenterology and Hepatology, Tianjin Union Medical Center, Tianjin Medical University, Tianjin, China; 3 Department of Gastroenterology and Hepatology, The Third Central Hospital of Tianjin, Tianjin, China; 4 Department of Hepatobiliary Surgery, The Third Central Hospital of Tianjin, Tianjin, China; 5 Tianjin Key Laboratory of Extracorporeal Life Support for Critical Diseases, Tianjin Institute of Hepatobiliary Disease, Nankai University Affinity the Third Central Hospital, Tianjin, China

**Keywords:** Cancer, Computational biology and bioinformatics

## Abstract

Bile acids and salts have been shown to play a role in liver carcinogenesis through DNA damage, inflammation, and tumor proliferation. However, the correlation between bile acid metabolism and hepatocellular carcinoma (HCC) prognosis remains unclear. This study aimed to identify a predictive signature of bile acid and bile salt metabolism-related long non-coding RNAs (lncRNAs) for HCC prognosis and treatment response. The study used HCC RNA-sequencing data and corresponding clinical and prognostic data from The Cancer Genome Atlas. A prognostic model consisting of five bile acid and bile salt metabolism-related lncRNAs was developed and evaluated in a training set, a validation set and an external set. The model demonstrated good performance in predicting HCC prognosis and was shown to be an independent biomarker for prognosis. Additionally, our study revealed a significant association between the signature and immune cell infiltration, as well as its predictive value for therapeutic responses to both immunotherapy and chemotherapy. Furthermore, three LncRNAs (LUCAT1, AL031985.3 and AC015908.3) expression levels in our signature were validated through qRT-PCR in a cohort of 50 pairs of HCC patient tumor samples and corresponding adjacent non-tumor samples, along with 10 samples of normal liver tissue adjacent to benign lesions. These findings suggest that this novel bile acid and bile salt metabolism-related lncRNA signature can independently predict the prognosis of patients with HCC and may be utilized as a potential predictor of response to treatment in this setting.

## Introduction

Liver cancer is one of the most common malignancies in humans and the second leading cause of cancer-related death worldwide^1,2^. Hepatocellular carcinoma (HCC) is the predominant form of liver cancer with a complex etiology and limited treatment options. As a result of the considerable efforts of researchers, various novel therapeutic methods are emerging. However, there is currently a lack of specific treatment. Recently, immunotherapy for advanced HCC has become a research hotspot. Unfortunately, the effectiveness of this treatment modality is limited^3^. Research on biomarkers of response or primary resistance to immunotherapies is essential for efficient treatment.

As endogenous metabolites of host-gut microflora co-metabolism, bile acids and their metabolites are attracting attention due to their carcinogenic potential. Bile acids, especially secondary bile acids, release arachidonic acid to promote the production of reactive oxygen species and induce DNA damage. Besides, bile acids and salts can also mediate inflammation, promote tumor proliferation and inhibit tumor cell apoptosis through a series of signal transduction pathways^4–6^. It has been shown that intestinal bacterial metabolite deoxycholic acid (DCA) promotes obesity-related liver cancer in mouse models^7^. In addition, the induction of inflammatory gene expression by DCA in hepatocytes was closely related to the occurrence and development of tumors^8^. Hang et al. suggested that bile acid metabolites directly regulate the balance between T-helper 17 and regulatory T cells to control host immune response without the involvement of intestinal flora^9^. Another study revealed that bile acids metabolized by intestinal flora can specifically regulate the number of natural killer T cells in the liver, thereby regulating the growth of liver tumors^10^. The above evidence clearly demonstrated that bile acids and salts play an important regulatory role directly or indirectly in shaping the immune system.

Long non-coding RNAs (lncRNAs) are non-coding transcripts with a length > 200 nucleotides, which regulate the expression of numerous cancer-related genes. An increasing number of studies have found that abnormally expressed lncRNAs are involved in regulating tumorigenesis-related biological functions, such as metastasis, immune response, and metabolic regulation^11,12^. In recent years, a growing body of evidence supports that many cellular actions of the bile acid/Farnesoid X receptor (FXR) pathway are mediated by lncRNAs, and lncRNAs are in turn powerful regulators of bile acid levels and FXR activities^13^. However, research on a bile acid and salt metabolism-related lncRNA prognostic signature in patients with HCC is currently lacking.

## Results

### Identification and visualization of differentially expressed bile acid and bile salt metabolism-related genes

**Figure 1:**
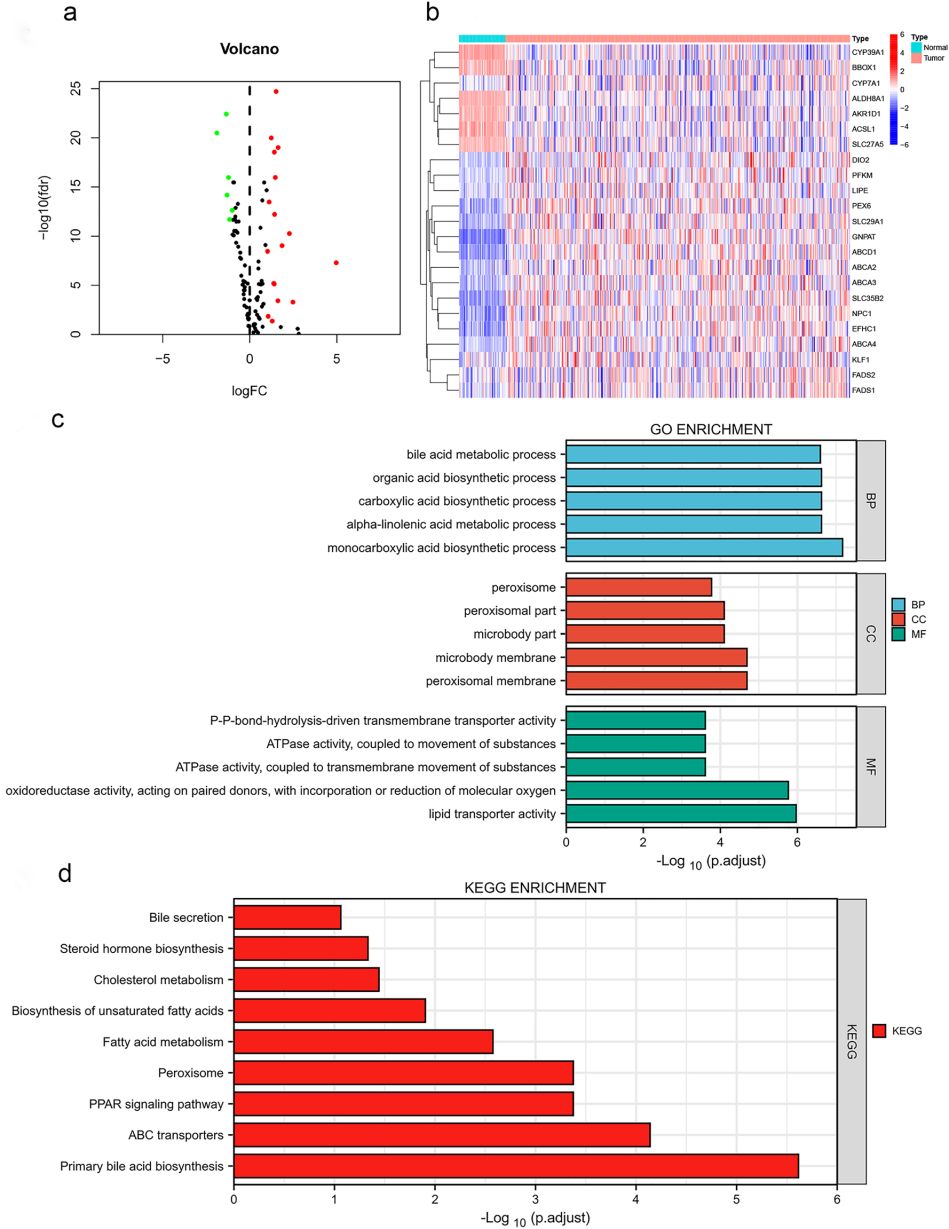
GO and KEGG analyses of 23 bile acid and salt metabolism-related DEGs in HCC (**a**) Volcano plot of bile acid and bile salt metabolism-related genes in HCC. Red and green dots represent upregulated and downregulated genes, respectively. (**b**) Heatmap showing the RNA expression levels of 23 DEGs. (**c**) GO analysis. (**d**) KEGG analysis. *ABC* ATP-binding cassette, *ATPase* adenosine triphosphatase, *BP* biological process, *CC* cellular components, *FC* fold change, *FDR* false discovery rate, *GO* Gene Ontology, *HCC* hepatocellular carcinoma, *KEGG* Kyoto Encyclopedia of Genes and Genomes, *MF* molecular function, *PPAR* peroxisome proliferator-activated receptor, *p.adjust* adjusted p value.

We obtained 23 bile acid and bile salt metabolism-related differentially expressed genes (DEGs), including 17 upregulated genes and and 6 downregulated genes (Fig. 1a, Supplementary Table S1). The heatmap shows the RNA expression levels of those DEGs (Fig. 1b). Gene Ontology (GO) and Kyoto Encyclopedia of Genes and Genomes (KEGG) analyses were performed to identify DEGs at the biologically functional level (Fig. 1c,d). In the biological process category, GO analysis showed that the DEGs were mainly enriched in the bile acid metabolic process, organic acid biosynthetic process, monocarboxylic acid biosynthetic process, etc. In the cellular components category, the DEGs were mainly enriched in peroxisome, peroxisomal part, peroxisomal membrane, microbody part, etc. In the molecular function category, the DEGs were mainly enriched in lipid transporter activity, oxidoreductase activity, acting on paired donors, with incorporation or reduction of molecular oxygen, etc. KEGG pathway analyses indicated that bile acid and bile salt metabolism-related DEGs were mainly enriched in the primary bile acid biosynthesis, ATP-binding cassette transporters, peroxisome proliferator-activated receptor (PPAR) signaling pathway, etc.

### Construction of a bile acid and bile salt metabolism-related lncRNA signature

**Figure 2:**
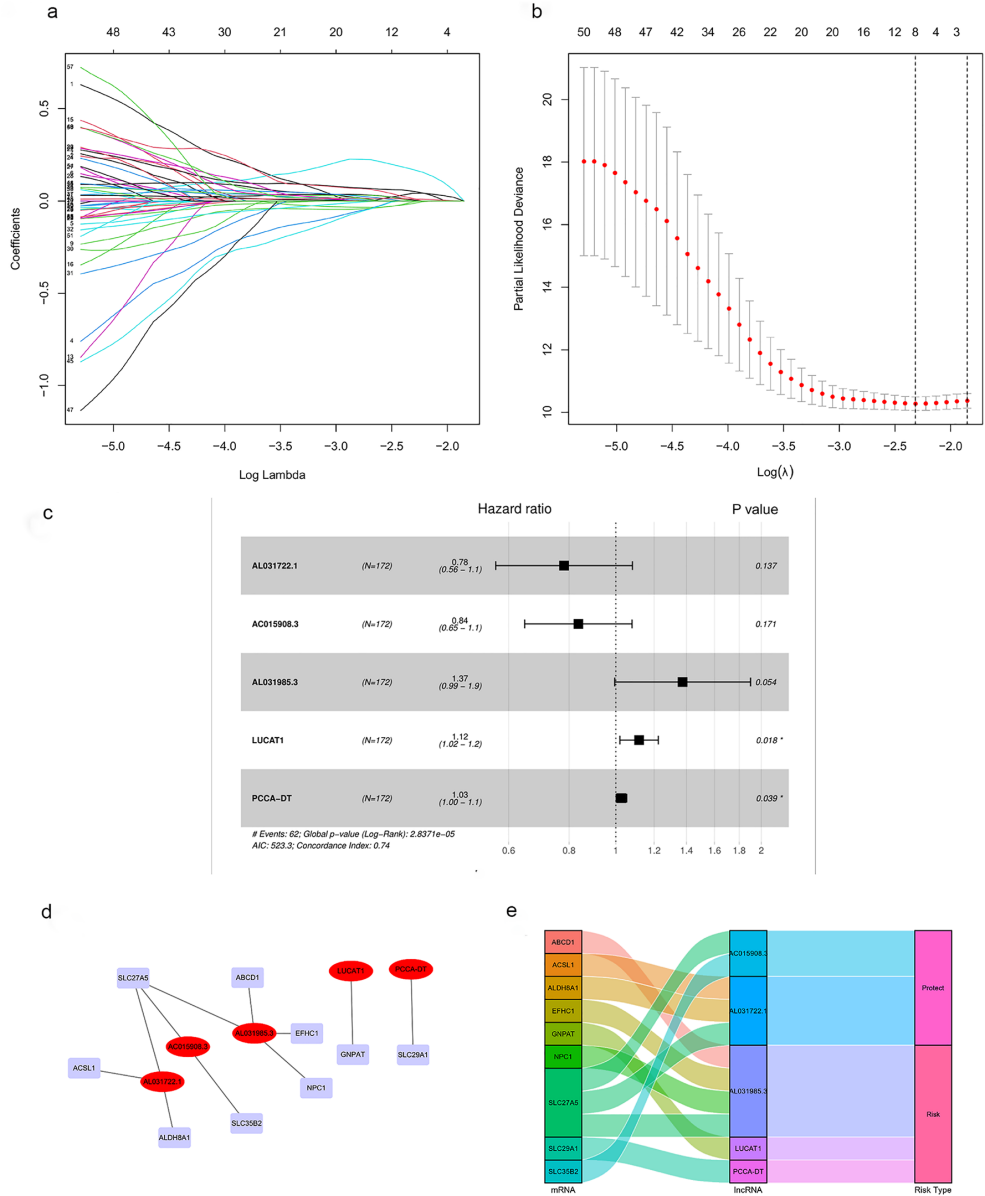
Regression coefficient diagram based on the LASSO algorithm and lncRNA-mRNA network. (**a**) LASSO coefficient profiles of 57 bile acid and bile salt metabolism-related lncRNAs. (**b**) Cross-validation for the selection of tuning parameter in the LASSO regression. (**c**) Forest plots showing the relationships of each lncRNA subset with overall survival in the training group. The unadjusted hazard ratios are presented with 95% confidence intervals. (**d**) The co-expression network of prognostic bile acid and bile salt metabolism-related lncRNAs. (**e**) Sankey diagram of prognostic bile acid metabolism-related lncRNAs. *LASSO* least absolute shrinkage and selection operator, *lncRNA* long non-coding RNA.

A total of 498 bile acid and salt metabolism-related lncRNAs were obtained by Pearson's correlation analyses. To construct a prognostic model for HCC, univariate Cox regression was performed, and 57 lncRNAs related survival were selected. Subsequently, analysis with the LASSO algorithm was performed to identify stable markers from the survival-related lncRNAs (Fig. 2a,b). Finally, multivariate cox regression analysis was performed and a prognostic lncRNA model consisting of five lncRNAs (i.e., AL031722.1, AC015908.3, AL031985.3, LUCAT1 and PCCA-DT) was constructed. The risk score was calculated as follows: risk score = AL031722.1 expression * (− 0.246917061576047) + AC015908.3 expression * (− 0.178616640082393) + AL031985.3 expression * 0.317991474159641 + LUCAT1 expression * 0.110874567602666 + PCCA-DT expression * 0.0266570817050312. In addition, the hazard ratios of the five lncRNAs were calculated. AL031722.1 and AC015908.3 were protective factors for prognosis, whereas the other three lncRNAs were risk factors (Fig. 2c). Furthermore, the lncRNA-mRNA co-expression network (Fig. 2d) was constructed using the five lncRNAs identified through Pearson correlation analysis (|r| > 0.3 and p < 0.001). A Sankey diagram was used to visualize the network (Fig. 2e).

### The predictive power of the prognostic model in patients with HCC

**Figure 3:**
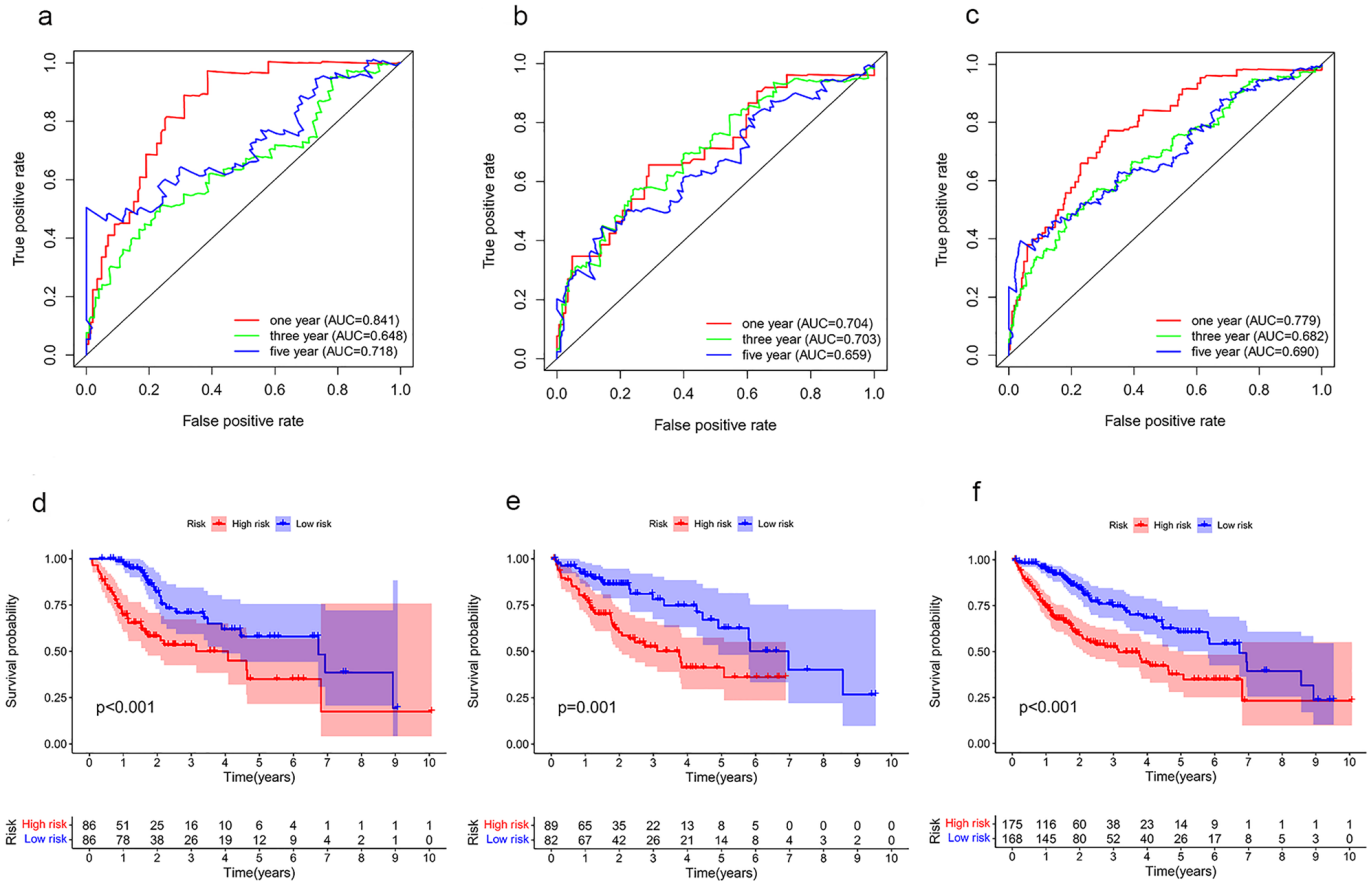
Internal validation of the predictive signature for OS based on the entire TCGA dataset (**a**–**c**) Time‐dependent ROC curves at 1, 3, and 5 years in training set, validation set and whole set. (**d**–**f**) The Kaplan–Meier survival curves of the bile acid and bile salt metabolism‐related lncRNA Signature in training set, validation set and whole set.

To evaluate the power of this bile acid and bile salt metabolism-related lncRNAs classifier in predicting the survival of patients with HCC, ROC analysis was performed in the training, verification and whole sets. The results showed that the area under the curve of the risk score at 1, 3 and 5 years of prognosis time in the training set was 0.841, 0.648, and 0.718, respectively. These findings indicated high sensitivity and specificity for the prediction of survival (Fig. 3a). The prognostic accuracy of this risk prediction model was further tested using an internal verification set and a whole set, which revealed good predictive performance (Fig. 3b,c).

**Figure 4:**
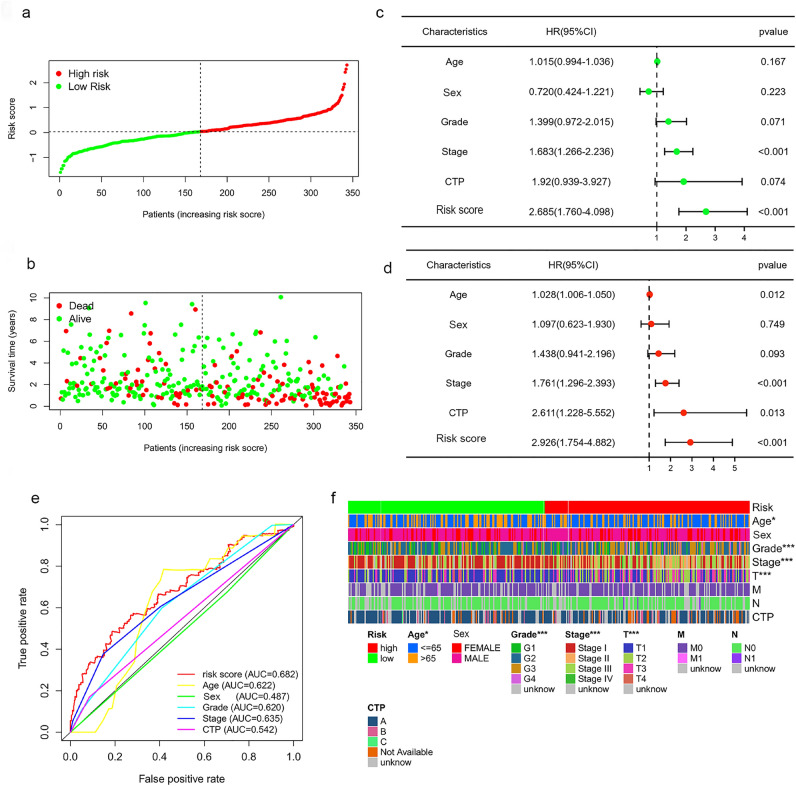
The correlation between the predictive signature and the prognosis of HCC patients. (**a**) Risk score curves based on the risk score of each patient with HCC. (**b**) Scatter plots displaying the survival status of each patient with HCC. (**c**) Forrest plot for the univariate Cox regression analysis of clinicopathological variables. (**d**) Forrest plot for the multivariate Cox regression analysis of clinicopathological variables. (**e**) The ROC curve of the risk score and clinicopathological variables. (**f**) Distribution heat map of clinicopathological variables in the low- and high-risk groups ***p < 0.001, **p < 0.01, *p < 0.05. *AUC* area under the curve, *CI* confidence interval, *CTP* Child–Turcotte–Pugh grade of liver function, *HR* hazard ratio, *ROC* receiver operating characteristic.

A prognostic curve and a scatter plot were used to exhibit the risk score and the survival status of each patient with HCC (Fig. 4a,b). The analysis showed that a high-risk score was associated with an increased mortality rate. In addition, Kaplan–Meier analysis was used to analyze the overall survival (OS) time in the low- and high-risk groups. Figure 3d–f showed that patients in the high-risk group had a worse prognosis than those in the low-risk group in the training, validation, and whole sets (all p < 0.05).

Cox regression analysis was performed to determine whether the risk score of the model is an independent prognostic factor for patients with HCC. Both univariate and multivariate Cox regression analyses showed that the risk score was significantly associated with the OS of patients with HCC (Fig. 4c,d). This observation indicated that the risk score was an independent factor for the prognosis of patients with HCC. We also compared several other clinical parameters to evaluate the important function of the risk score in predicting prognosis. Age, sex, stage, grade, and Child–Turcotte–Pugh (CTP) grade of liver function were examined as candidate predictive biomolecular indicators. The area under the curve of the risk score was 0.682, which was better than those of other clinicopathological variables in predicting the prognosis of patients with HCC (Fig. 4e). Figure 4f presents a heatmap displaying the distribution of clinicopathological variables in the high- and low-risk groups. The data reveal significant dissimilarities (all p < 0.05) between the two groups in terms of T stage, stage, grade, and age.

### Construction and verification of the prognostic nomogram

**Figure 5:**
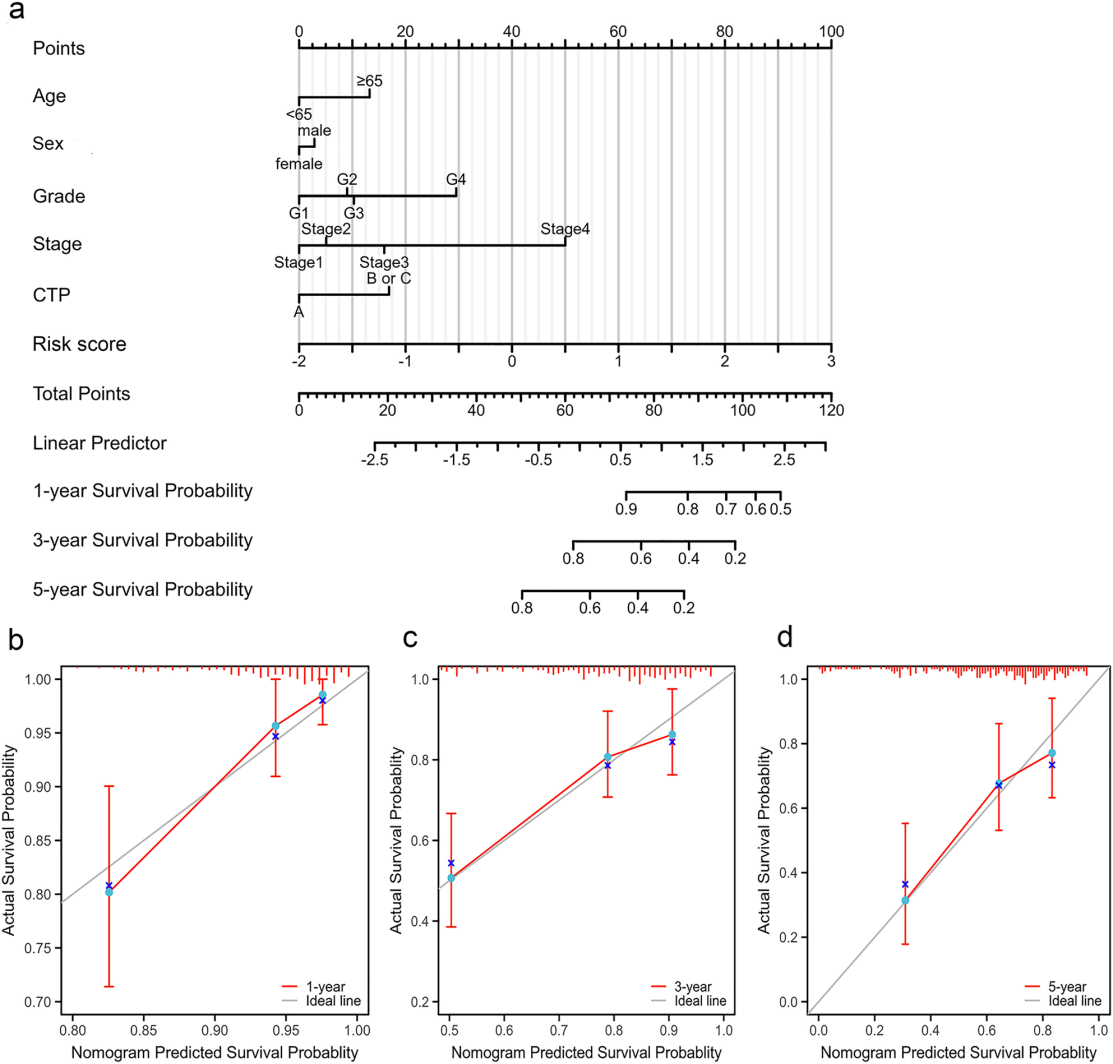
Construction and verification of the nomogram. (**a**) A nomogram combining clinicopathological parameters and the risk score for estimating the probability of 1-, 3-, and 5-year survival of patients with HCC. (**b**–**d**) Calibration curves were used to investigate the deviation between the predicted and actual survival rates at 1, 3, and 5 years. *CTP* Child–Turcotte–Pugh grade of liver function, *HCC* hepatocellular carcinoma.

To better evaluate the predictive power of this model for survival, we constructed a nomogram based on the age, sex, CTP grade of liver function, grade, stage, and risk score (Fig. 5a). In addition, calibration plots were produced to evaluate the consistence between the nomogram-predicted and actual 1-, 3-, and 5-year survival rates. The predicted lines were basically consistent with the reference lines, indicating that the constructed nomogram was credible (Fig. 5b–d).

### Validation of bile acid and bile salt metabolism-related risk model in external cohorts

**Figure 6:**
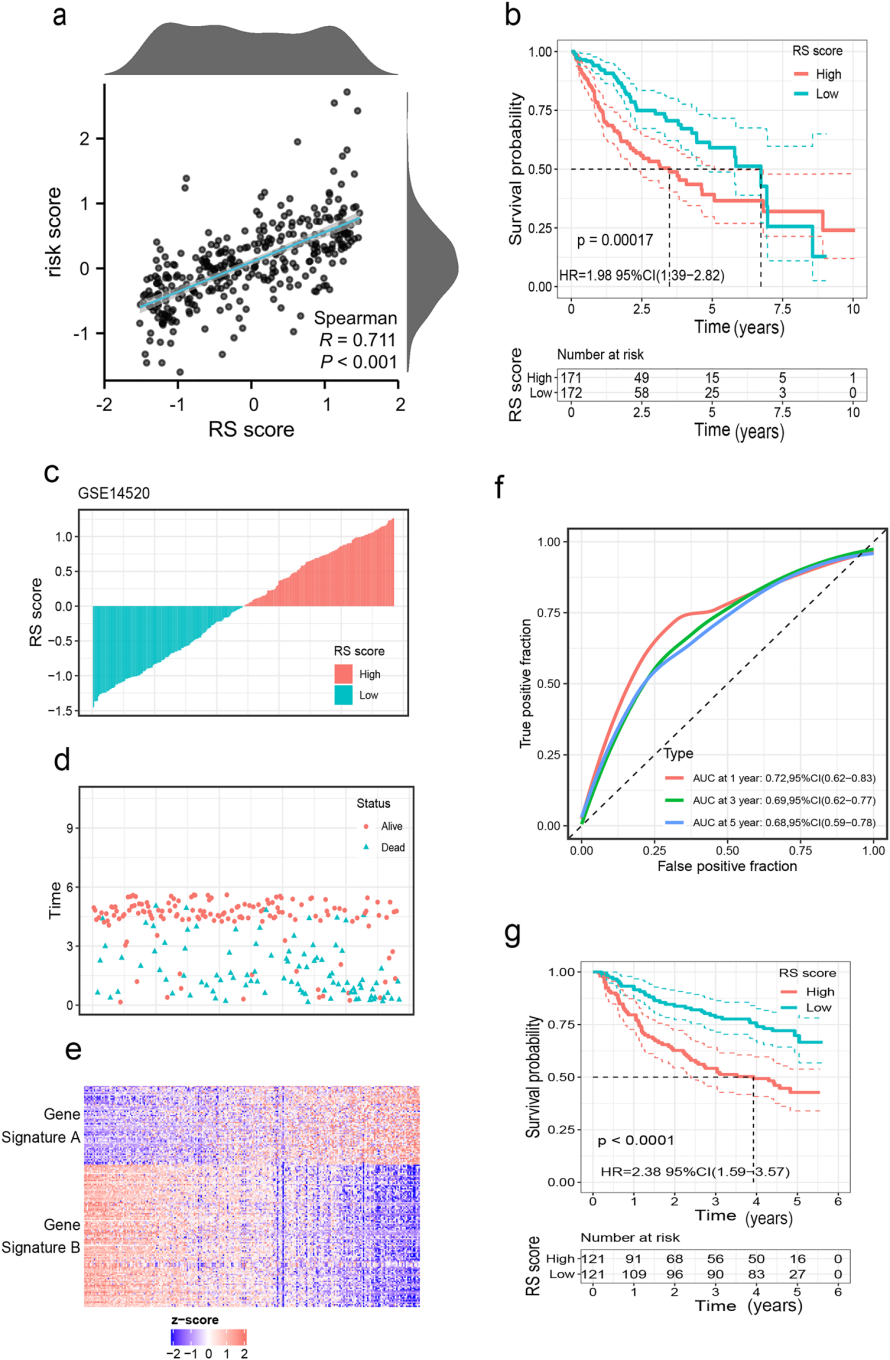
Construction of the RS score and validation in the external data set GSE14520. (**a**) Spearman correlation analysis between RS score and risk score. (**b**) The Kaplan–Meier curve shows the OS of patients with different RS scores in the TCGA-LIHC database. (**c**) Distribution plots for the relationship between RS score and survival status (**d**) Scatter plots displaying the survival status of each patient with HCC (**e**) Heat maps for the gene signature A/B in the cohort (**f**) ROC curve for the RS score in the external cohort (**g**) Survival curves between high-and low-RS score groups in GSE14520.

Based on the risk model, we identified a differentially expressed gene signature A/B between the low-risk and high-risk groups within the TCGA-LIHC dataset. The Risk Gene Signature Score (RS score) was calculated based on the expression differences between gene signatures A and B in these high- and low-risk groups. In the TCGA-LIHC database, a significant correlation was observed between the risk score derived from bile acid metabolism-related lncRNAs and the RS score (as shown in Fig. 6a). Kaplan–Meier analysis further confirmed that high RS scores were associated with poor prognosis (Fig. 6b). These results collectively indicate that the RS score method represents a viable alternative risk score model.

In the GSE14250 dataset, we applied the same RS score calculation method to each patient. Patients were then divided into high-RS score and low-RS score groups based on the calculated median RS score. Survival analyses conducted on this external cohort consistently demonstrated that patients with a high RS score had an unfavorable prognosis (Fig. 6g). Additionally, ROC curve analysis illustrated the reliable prognostic predictive ability of the alternative model (Fig. 6f).

### Gene set enrichment analysis (GSEA) in the low- and high-risk groups

GSEA was used to annotate the biological functions and determine the enrichment pathways in the low- and high-risk groups. In the high-risk group, there were 87 significantly enriched pathways (Supplementary Table S2), mainly related to proliferation, apoptosis, tumors, and other related pathways (Supplementary Fig. S1). In the low-risk group, there were 38 significantly enriched pathways (Supplementary Table S3), mainly related to metabolism pathways, such as primary bile acid biosynthesis, fatty acid metabolism, glycine serine and threonine metabolism, etc. (Supplementary Fig. S1).

### The predictive signature and immune cell infiltration

**Figure 7:**
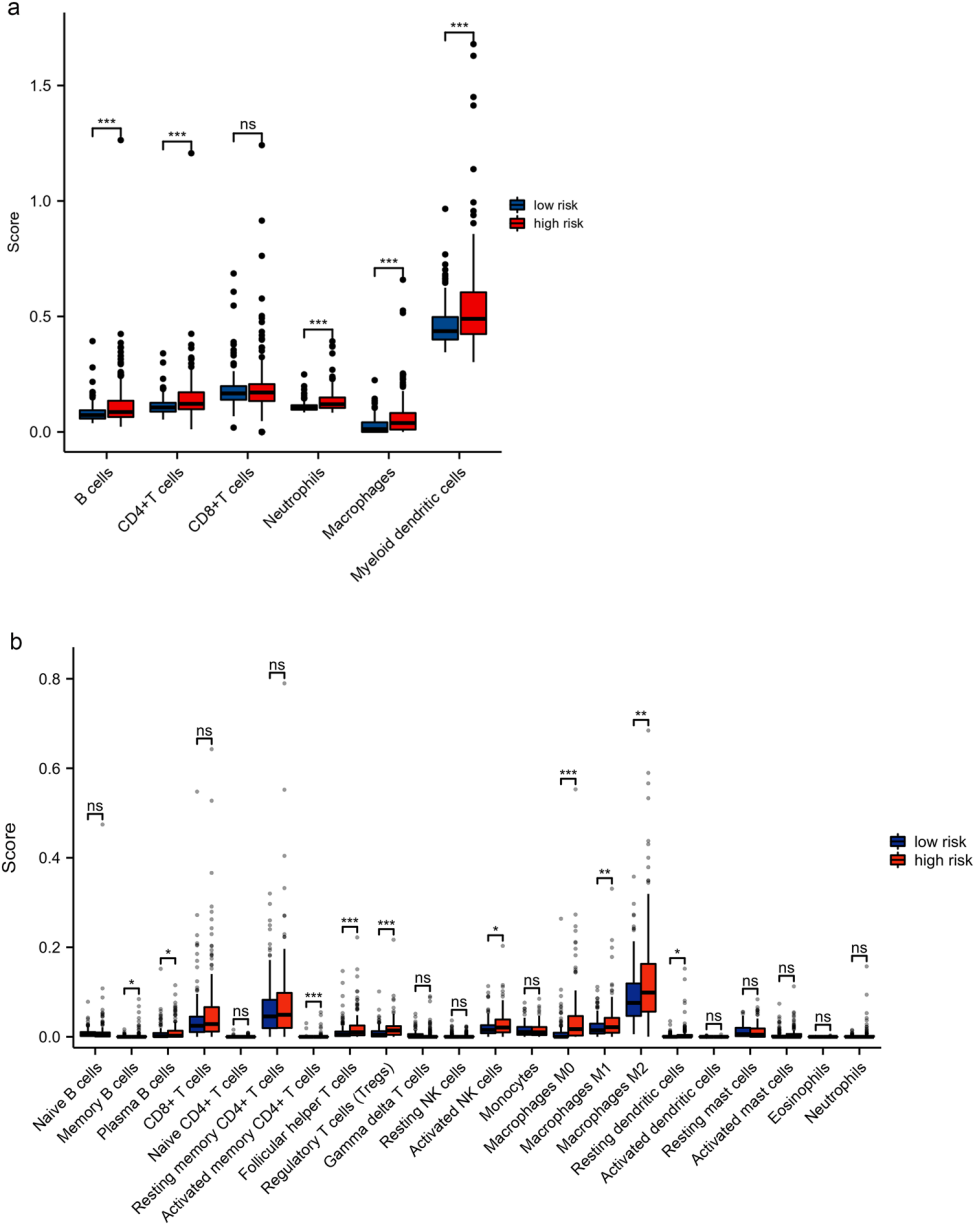
Immune cell infiltration in the low- and high-risk groups. (**a**) The TIMER algorithm was used to calculate the infiltration of six kinds of immune cells. (**b**) The CIBERSORT abs.mode algorithm was used to calculate the infiltration of 22 kinds of immune cells (Wilcoxon rank-sum test, ns, not statistically significant; p ≥ 0.05; *p < 0.05; **p < 0.01; ***p < 0.001). *TIMER* tumor immune estimation resource.

To evaluate the association between our lncRNA signature and immune infiltration, we drew the correlation bubble plot of immune cells and risk score calculated using various algorithms (Supplementary Fig. S2). From the figure, it can be inferred that the scores of most immune cells were positively correlated with the risk score. In addition, the TIMER and CIBERSORT abs.mode algorithms were applied to evaluate the infiltration of immune cells in the low- and high-risk groups. The TIMER algorithm yielded six kinds of immune cells in total. The immune scores of B cells, CD4 + T cells, neutrophils, macrophages, and myeloid dendritic cells were higher in the high-risk group versus the low-risk group (Fig. 7a). The CIBERSORT abs.mode algorithm calculated the immune scores of 22 types of immune cells. It was found that numerous cell types (i.e., memory B cells, plasma B cells, activated memory CD4 + T cells, follicular helper T cells, regulatory T cells, activated NK cells, resting dendritic cells, M0 macrophages, M1 macrophages and M2 macrophages) had a higher immune score in the high-risk group versus the low-risk group (Fig. 7b). We also use ESTIMATE algorithm^14^ to evaluate the relationship of the risk score and the ratio of immune score in tumor samples. It was found that immune score was higher in the high-risk group (p = 0.037, Fig. 9b), which indicated that the high-risk group samples might had a higher ratio of immune cells.

### Correlation between the predictive signature and immune checkpoint blockade (ICB) therapy-related molecules

**Figure 8:**
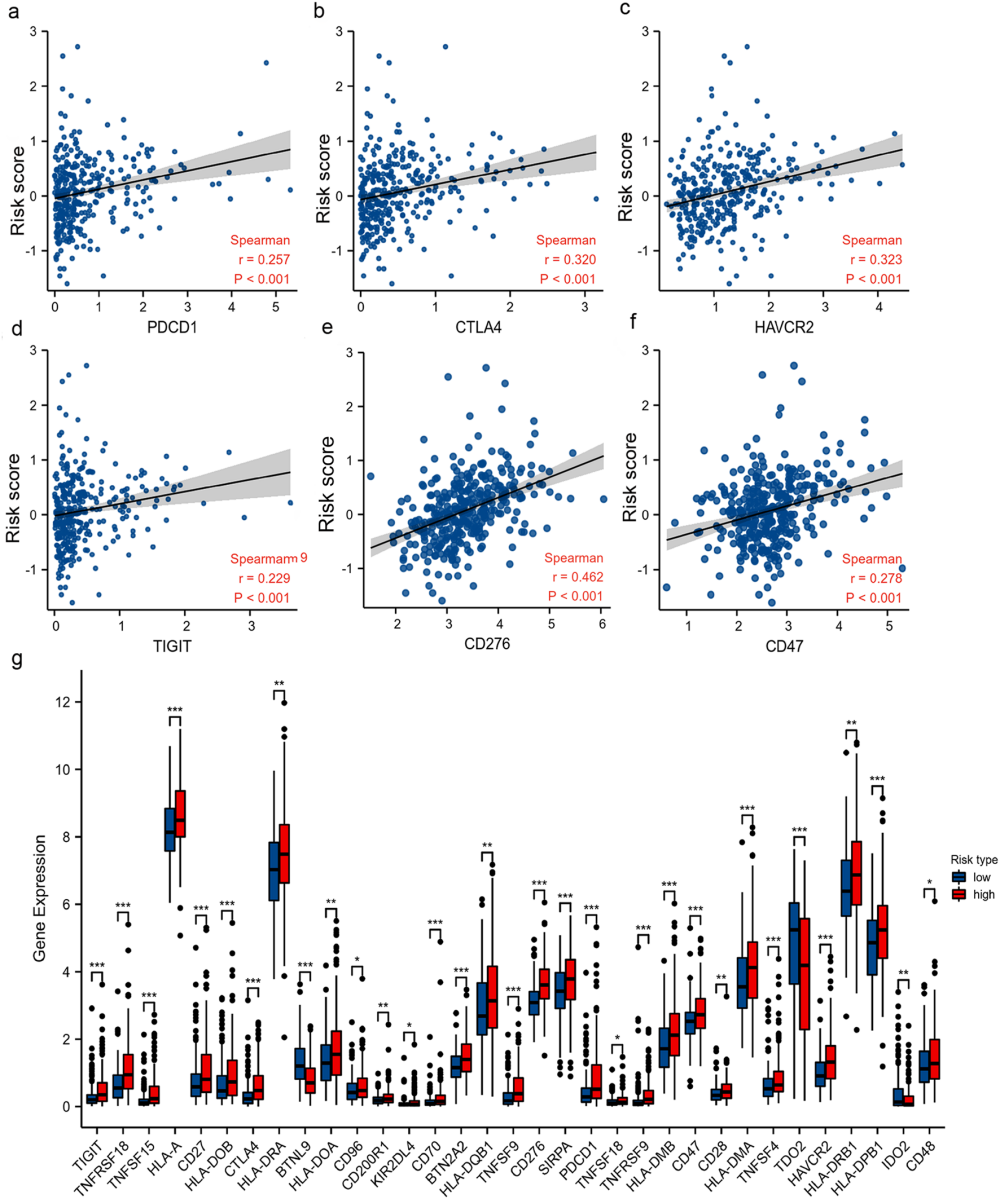
Correlation between the expression of ICB-related genes and this bile acid and bile salt metabolism-related lncRNA signature. (**a**–**f**) Association between the risk score of this signature and the expression of six ICB-related genes (i.e., PD-1, CTLA4, HAVCR2, TIGIT, CD276 and CD47) (**g**) Comparison of 33 differentially expressed ICB-related genes in the low- and high-risk groups (Wilcoxon rank sum test, ns, not statistically significant; *p < 0.05; **p < 0.01; ***p < 0.001). *CD47* cluster of differentiation 47, *CD276* Cluster of differentiation 276, *CTLA4* cytotoxic T-lymphocyte associated protein 4, *ICB* immune checkpoint blockade, *lncRNA* long non-coding RNA, *PDCD1* programmed cell death 1, *TIGIT* T Cell immunoreceptor with Ig and ITIM domains.

To explore the role of the bile acid and salt metabolism-related lncRNA risk score model in ICB immunotherapy in patients with HCC, we analyzed the correlation between key targets of ICB therapy and this lncRNA signature. We compared the expression levels of 47 immune checkpoint genes (Supplementary Table S4) in the low- and high-risk groups. We found that the expression levels of 33 genes exhibited statistically significant differences; most of them were highly expressed in the high-risk group (Fig. 8g). To further clarify the relationship between our bile acid and salt metabolism-related lncRNA model and immune checkpoint genes, we analyzed six immune checkpoint genes. Spearman analysis was used to determine the correlation between the expression levels of these genes and the risk scores of the model (Fig. 8a–f). The results showed that the risk score was significantly positively correlated with PD-1 (r = 0.257, p < 0.001), CTLA4 (r = 0.320, p < 0.001), TIM-3 (r = 0.323, p < 0.001), TIGIT (r = 0.229, p < 0.001), CD276 (r = 0.462, p < 0.001), and CD47 (r = 0.278, p < 0.001).

### Correlation between the predictive signature and therapy response in HCC

**Figure 9:**
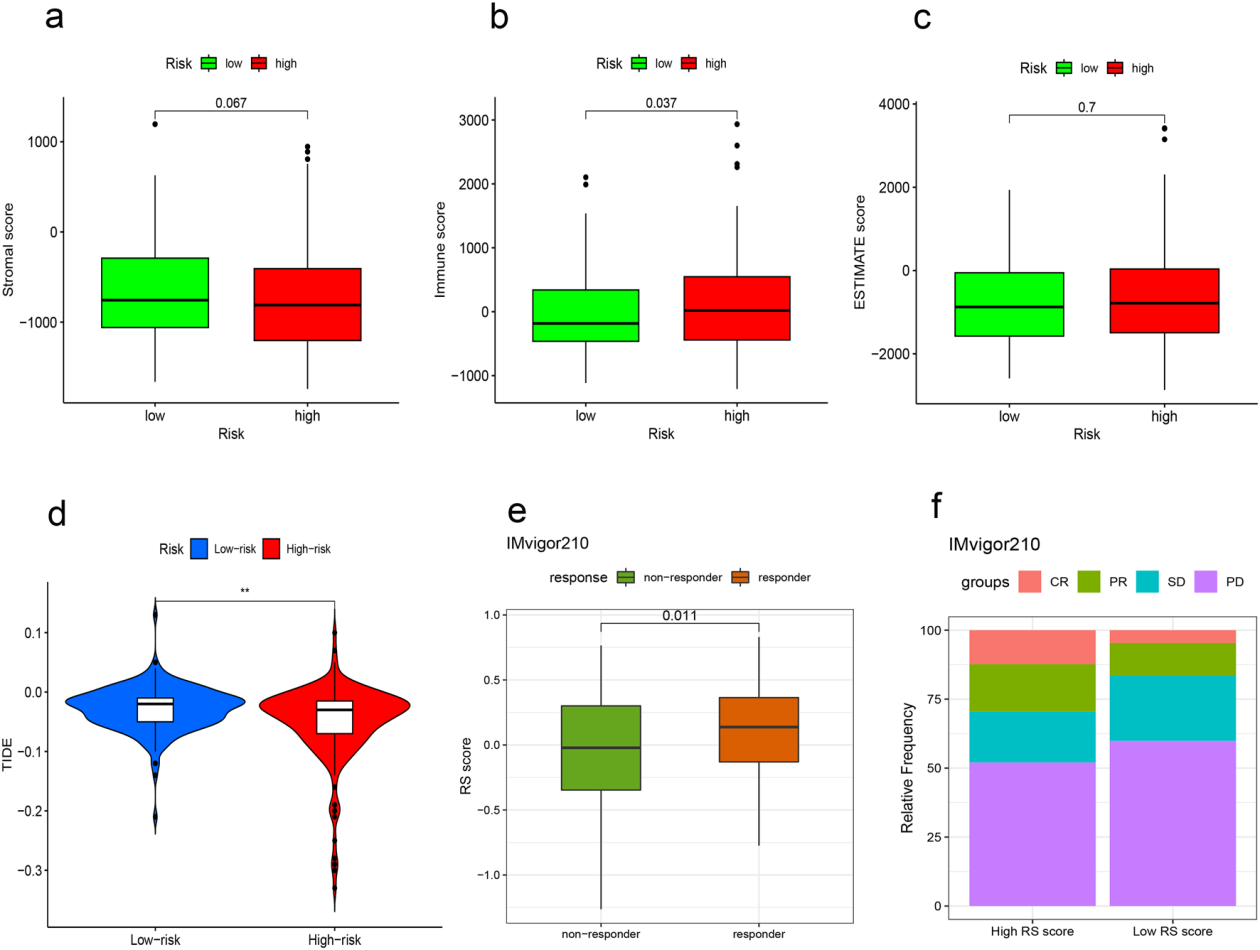
Differences in immune infiltration and immune therapy response between high- and low-risk groups. (**a**) Comparison of the Stromal scores between low- and high-risk group using the ESTIMATE algorithm. (**b**) Comparison of the Immune scores between low- and high-risk group. (**c**) Comparison of the Estimate scores between low- and high-risk group. (**d**) Comparison of the TIDE scores between low- and high-risk group. (**e**) Boxplot demonstrating the RS score difference between the response group and the non-response group in IMvigor210 dataset. (**f**) Bar plot displaying the relative frequency of different clinical response subgroups in the low or high RS score group in IMvigor210 dataset.

We further evaluated whether the bile acid and salt metabolism related signature could serve as an immunotherapy predictor for HCC patients based on the tumor immune dysfunction and exclusion (TIDE) algorithm. Higher TIDE prediction scores are associated with a greater likelihood of immune evasion, indicating that patients are less likely to benefit from ICI treatment. Interestingly, our study revealed significantly lower TIDE scores in high-risk group compared to the low-risk group (p < 0.01, Fig. 9d), implying that high-risk patients may derive more substantial benefits from ICI therapy. Furthermore, through an analysis of the IMvigor210 immunotherapy dataset, we observed that patients with high RS Scores (equivalent to a high-risk profile) exhibited a notably higher response rate to immunotherapy (p = 0.011). This finding further substantiates the notion that high-risk group patients stand to gain greater benefits from immunotherapy.

**Figure 10:**
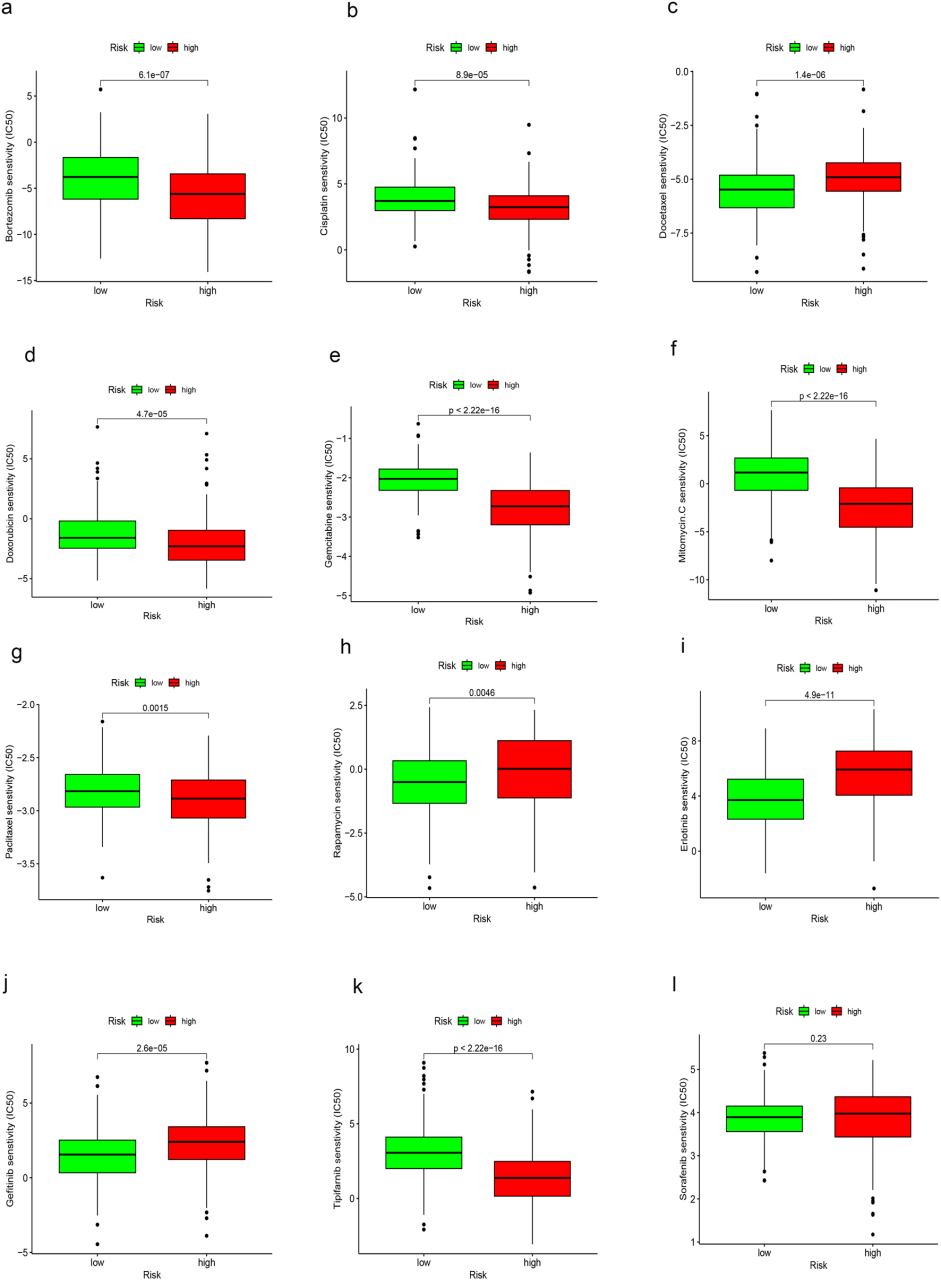
Comparison of the sensitivity of the low- and high-risk groups to chemotherapeutic and targeted drugs. (**a**–**h**) Estimated IC_50_ of chemotherapeutic drugs Bortezomib, Cisplatin, Docetaxel, Doxorubicin, Gemcitabine, Mitomycin.C, Paclitaxel and Rapamycin in the low- and high-risk groups. (**i**–**l**) Estimated IC_50_ of targeted drugs Erlotinib, Gefitinib, Tipifarnib and Sorafenib in the low- and high-risk groups. *IC*_*50*_ half-maximal inhibitory concentration.

The "pRRophetic" package of the R project was used to predict the sensitivity of patients to different chemotherapeutic and targeted drugs using the risk score^15,16^ (Fig. 10). We found that low-risk group was associated with the low estimated IC_50_ of Docetaxel and Rapamycin, and high-risk group was associated with the low estimated IC_50_ of Bortezomib, Cisplatin, Doxorubicin, Gemcitabine, Mitomycin.C and paclitaxel (Fig. 10a–h). We also found the estimated IC_50_ of some targeted drugs like Erlotinib and Gefitinib were lower in low-risk group, and Tipifarnib was lower in high-risk group (Fig. 10i–k). However, there was no significant difference in estimated IC_50_ between high- and low-risk groups for Sorafenib (Fig. 10l). These findings suggested that the risk score can be used as a predictor of sensitivity to chemotherapy.

### qRT-PCR validation of liver cancer samples

**Figure 11:**
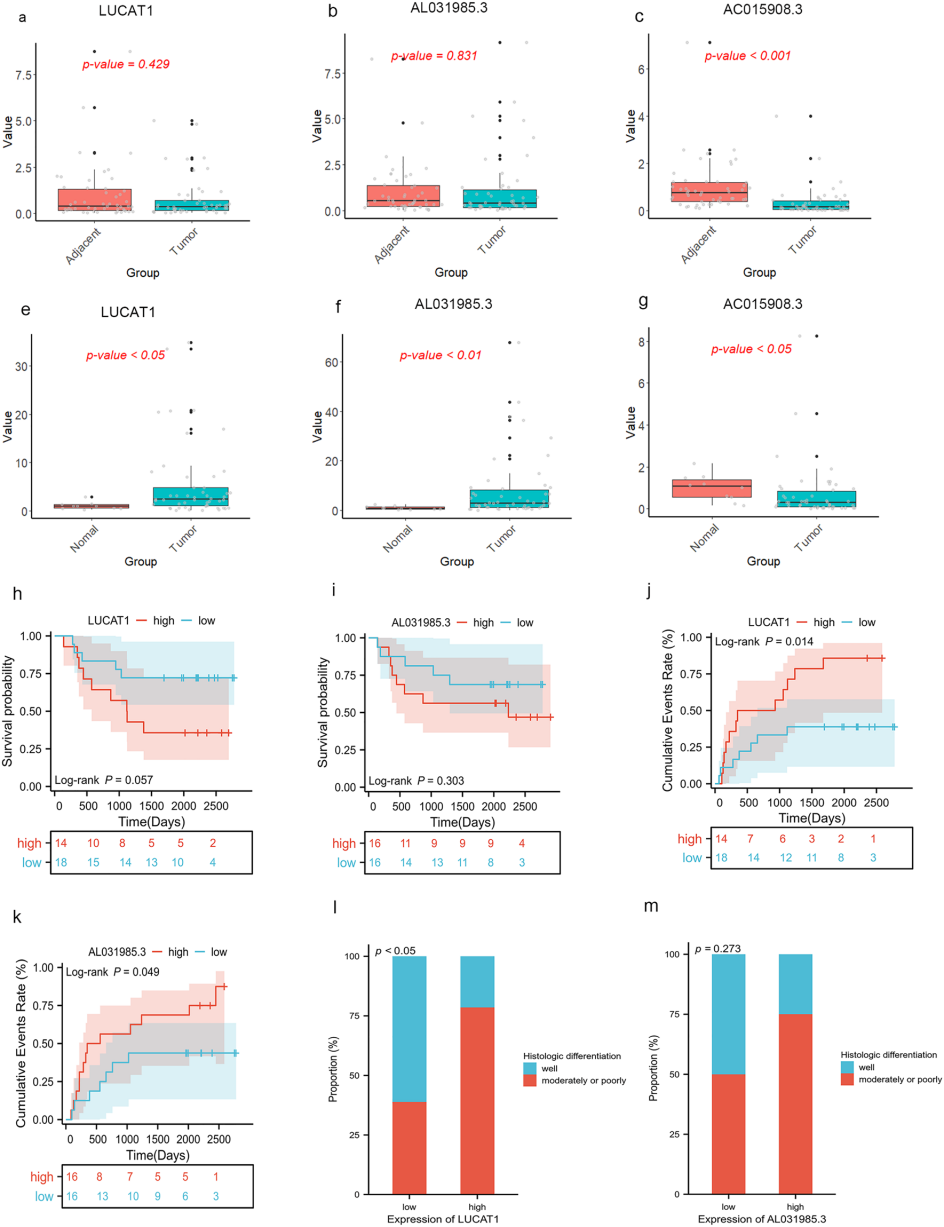
Validation of lncRNA expression by qRT-PCR in clinical samples and exploration of their association with clinical features. (**a**–**c**) Expression differences of LUCAT1, AL031985.3, and AC015908.3 in Hepatocellular Carcinoma (HCC) and corresponding adjacent non-tumor tissues. (**d**–**f**) Expression differences of LUCAT1, AL031985.3, and AC015908.3 in HCC and normal liver tissues. (**h**, **i**) Kaplan–Meier survival curves comparing high and low-expression groups of LUCAT1 and AL031985.3. (**j**, **k**) Cumulative incidence of recurrence curves for high and low-expression groups of LUCAT1 and AL031985.3. (**l**, **m**) Overlayed bar graphs displaying the differences in Histologic differentiation between high and low-expression groups of LUCAT1 and AL031985.3.

We collected tissues and corresponding clinical prognostic information from 50 patients with HCC. Initially, the expression differences of three lncRNAs in tumor samples and adjacent non-tumor samples were compared. Through PCR analysis, we found that AC015908.3 was highly expressed in adjacent non-tumor tissues but exhibited low expression in tumor tissues (p < 0.001, Fig. 11c). However, the expression differences of LUCAT1 and AL031985.3 between tumor and adjacent non-tumor tissues were not found to be statistically significant (p values were 0.429 and 0.831, respectively, Fig. 11a,b). Subsequently, the expression of these three lncRNAs in tumor tissues was compared to that in normal liver tissues. It was found that both LUCAT1 and AL031985.3 were upregulated in tumor tissues compared to normal tissues (p values < 0.05 for both, Fig. 11e,f), while AC015908.3 exhibited higher expression in normal liver tissues and lower expression in tumor tissues (p < 0.04, Fig. 11g). To further investigate the clinical implications, stratified analyses were performed on LUCAT1 and AL031985.3. The high-expression group in tumor samples was defined as those with expression levels at least twice as high as in adjacent non-tumor samples, and the low-expression group as those with expression levels at half or less. Others were considered to have no significant change in expression. The LUCAT1 high-expression group comprised 14 cases, while the low-expression group included 18 cases. Similarly, the AL031985.3 high-expression group had 16 cases, and the low-expression group had 16 cases. The relationship between high and low-expression groups and clinical pathological features was explored. It was found that, for LUCAT1, there was no statistically significant difference in terms of overall survival (p = 0.057, Fig. 11h). However, a significantly higher cumulative recurrence rate was observed in the high-expression group compared to the low-expression group (p = 0.014, Fig. 11j). Additionally, a higher proportion of cases with moderately to poorly histological differentiation was found in the high-expression group compared to the low-expression group (p < 0.05, Fig. 11l). In the case of AL031985.3, there were no statistically significant differences in terms of overall survival or histological differentiation (Fig. 11j,l). However, a higher recurrence rate was observed in the high-expression group compared to the low-expression group (p = 0.049, Fig. 11k).

Comparisons between HCC and adjacent non-tumor tissues were assessed using the Wilcoxon matched-pairs signed rank test. Comparisons between HCC and normal liver tissues were conducted using the Wilcoxon rank-sum test. Survival and cumulative recurrence curves were compared using the log-rank test. Inter-group differences in histological frequencies were evaluated using Fisher's exact test.

## Discussion

Bile acids are synthesized in the liver and transformed into bile salts by the association with Na^+^ or K^+^ ions before release into the gall bladder. These primary bile acids are metabolized into secondary bile acids by certain members of the normal gut microbiota. Bile salt metabolism is a subset of bile acid metabolism that specifically focuses on the modified and more water-soluble forms of bile acids, known as bile salts. Bile acids and salts play important roles in the maintenance of the healthy gut flora, balance of lipid and carbohydrate metabolism, insulin sensitivity, and innate immunity. Moreover, as signaling molecules, they also play a pivotal role in the development of various types of cancer^17–20^. Accumulating evidence indicates that hydrophobic bile acids (e.g., lithocholic acid, DCA and chenodeoxycholic acid) may contribute to the tumorigenesis and development of liver cancer^21–23^. Huang et al. identified six microbial markers, which are closely related to bile acid metabolism and the tumor immune microenvironment, by fecal 16sRNA sequencing of patients with HBV-RELATED HCC and healthy populations. These markers had good prediction potential for clinical outcomes of HBV-related HCC patients^24^. FXR activity is a major inhibitor of HCC carcinogenesis. Dysregulation of bile acid homeostasis in cirrhosis and non-alcoholic steatohepatitis leads to increased hepatic bile acid inflammation and reduced FXR signaling, which is a risk factor for the development of HCC^2,25,26^. The sodium taurocholate cotransporting polypeptide (NTCP), which is involved in bile acid transport, has been identified as a receptor for HBV, and its variant, S267F, has been strongly associated with a decrease in HBV-associated hepatocellular carcinoma^27,28^. Petrick et al. provided evidence that higher concentrations of bile acids-specifically, conjugated primary bile acids-are associated with increased HBV- and HCV-related HCC risk^29^. In recent years, bile acid-related lncRNAs have been increasingly recognized as essential in the pathogenesis of liver diseases, including liver injury, fatty liver, fibrosis, and hepatocarcinoma^30–33^. However, few studies have described the role of bile acid metabolism in the prognosis of HCC. A comprehensive bioinformatics analysis is needed to evaluate the role of bile acid regulators in predicting prognosis and their therapeutic potential in HCC.

In this study, we identified 23 bile acid metabolism-related DEGs of HCC. KEGG analysis showed that the DEGs were mainly enriched in bile acid synthesis, metabolism, transport, and the PPAR signaling pathway. Numerous studies have shown that abnormal regulation of the PPAR signaling pathway is involved in the development and progression of tumors^34–36^. However, further experiments are needed to verify whether bile acid metabolism-related genes are involved in tumorigenesis and development through the PPAR signaling pathway in HCC.

Through stepwise univariate Cox regression, LASSO regression and multivariate Cox regression analyses, we constructed a novel bile acid and bile salt metabolism-related five-lncRNA signature in a large-scale HCC cohort. The results demonstrated the sensitivity and specificity of the signature. Subsequently, Kaplan–Meier curves, time-dependent ROC curves, and Cox regression analysis were employed to confirm the predictive performance of this bile acid metabolism-related lncRNA risk score model, which can serve as an independent biomolecular indicator for the prediction of patient survival in HCC. Furthermore, the calibration curves of the nomogram validated that our novel risk score model performs better than traditional clinicopathological characteristics in predicting the prognosis of HCC.

GSEA analysis showed that pathways enriched in the high-risk group were mainly related to proliferation, apoptosis and tumors; these pathways included Cell cycle, Oocyte meiosis, p53 signaling pathway, mTOR signaling pathway and Bladder cancer. Pathways enriched in the low-risk group were primarily involved in synthetic and metabolic processes, such as primary bile acid biosynthesis, fatty acid metabolism, and glycine serine and threonine metabolism. This suggests that more cancer-driving pathways (e.g. tumor cell proliferation, growth, differentiation and metabolism) are involved in the high-risk group versus the low-risk group, thus resulting in a worse prognosis. Metabolic reprogramming is an important feature of neoplasms and mutually causal in the occurrence and development of tumors. Hence, cancer is a genetic disease as well as a metabolic disease^37^. From the enrichment of normal metabolic pathways in the low-risk group and the enrichment of cancer-driving pathways in the high-risk group, it can be hypothesized that the five lncRNAs identified in this study may play a role in changes in the metabolic pathway, leading to the progression of HCC.

ICB therapy has revolutionized the treatment of numerous malignancies. Nevertheless, its effectiveness is limited to a minority of patients with cancer. Hence, the discovery of predictive and prognostic biomarkers is urgently warranted. However, currently, there is no effective biomarker for predicting the response of patients with HCC to treatment with immune checkpoint inhibitors^38^. In our study, we explored the relationship between our predictive signature and immune cell infiltration. As an integral component of the tumor microenvironment, immune infiltrates contribute to tumor progression and response to immunotherapy^39^. In some solid tumors, high levels of immune infiltration involving macrophages and T cells are usually associated with better immune response compared with low levels of immune infiltration^40^. In addition to T cells which have been extensively studied, other immune cells of the innate and adaptive immune systems, including dendritic cells (DCs), macrophages, NK cells, and B cells, have also been shown to contribute to tumor progression and immunotherapy response^41^. Hollern et al. demonstrated that immune checkpoint therapy induces T follicular helper cell (TFH cells) activation of B cells to facilitate the anti-tumor response in mouse models of triple-negative breast cancer^42^. An extensive analysis of 70,000 cancer patients across various types identified immune factors such as CD8 + T cells, M1 macrophages, TLSs, TH1 cells, TFH cells, B cells, NK cells, and DCs that were associated with favorable prognosis for most cancers analyzed. On the contrary, regulatory T cells (Treg cells), M2 macrophages, T-helper 17 cells (TH17), T-helper 2 cells (TH2), and polymorphonuclear myeloid-derived suppressor cells (PMN MDSCs) predominantly signaled adverse prognosis^43^. However, various immune cells play a dual role in tumor initiation and progression. Immune cells such as macrophages, dendritic cells, NK cells, B cells, CD4 + T cells, and CD8 + T cells can bolster anti-tumor immune responses, shielding us against malignant cells. Nonetheless, they can also adopt a pro-tumorigenic role, thereby fostering tumor advancement and survival^44^.

In our study, most immune cells had a positive correlation with the risk score; five of the six types of immune cells identified by the TIMER algorithm were enriched and had infiltrated in the high-risk group. Based on the results obtained from the CIBERSORT abs.mode algorithm analysis, there were significant differences in immune cells (e.g. memory B cells, plasma B cells, activated memory CD4 + T cells, follicular helper T cells, regulatory T cells, activated NK cells, resting dendritic cells and macrophages) between the low- and high-risk groups. These cells exhibited higher levels of infiltration in the high-risk group versus the low-risk group. And the ESTIMATE algorithm revealed that immune score was higher in the high-risk group than low-risk group, which means high-risk patients had higher infiltration of immune cells than low-risk patients. Subsequently, the relationship between the bile acid and salt metabolism-related lncRNA risk model and 47 ICB-related genes was explored. We found that 33 genes were differentially expressed in the low- and high-risk groups; most of these genes were highly expressed in the high-risk group. Spearman correlation analysis showed that the risk score of our bile acid and bile salt metabolism related-lncRNAs model was significantly correlated with the ICB-related genes (i.e., PD-1, TIM-3, and CTLA4). We found that high risk scores were linked to high expression of immune checkpoints. This suggested that bile acid metabolism is important in the field of immuno-oncology. To ascertain the association of our model with the benefit of immunotherapy response, we independently validated the immune therapy response in high and low-risk groups using the TIDE database and the immunotherapy cohort IMvigor 210. The TIDE scores were lower in the high-risk group, indicating that there is a reduced likelihood of immune evasion in this group, suggesting that they may derive greater benefits from immunotherapy. Furthermore, due to the lack of expression data for the five lncRNAs in our model, we used the RS score as a substitute for the risk score and observed that higher RS scores were linked to improved immune responses in the IMvigor 210 cohort. In summary, it can be inferred that patients in the high-risk group may potentially benefit more from immunotherapy compared to those in the low-risk group. We also assessed the role of this model in predicting the response to conventional chemotherapeutic drugs used in HCC. By calculating the estimated IC_50_ values, we found that high-risk patients may be sensitive to cisplatin, mitomycin C and doxorubicin, but resistant to docetaxel. Based on our study, the use of immunotherapy or chemotherapeutic drugs can be customized to individual patients according to their risk score and sensitivity, thereby providing the basis for precise and personalized treatment in HCC. However, additional laboratory experiments and large-scale clinical trials are warranted in the future to confirm this hypothesis.

In our study, we conducted qRT-PCR to assess the expression levels of three lncRNAs included in our model in HCC tissues. We found that LUCAT1 and AL031985.3 exhibited higher expression levels in tumor tissues compared to normal liver tissues, while AC015908.3 showed lower expression in tumor tissues compared to normal liver tissues. These findings were consistent with the results obtained from mining the TCGA-LIHC database. Increasing evidence have showed that lncRNA lung cancer-associated transcript 1 (LUCAT1) participates in the regulation of proliferation, migration, invasion, and drug resistance of multiple tumors^45–48^. LUCAT1 exhibits high expression in various types of cancers. In breast cancer, elevated LUCAT1 expression is associated with poorer survival, larger tumor size, and later TNM staging^46^. In colorectal cancer, LUCAT1 promotes cell proliferation, apoptosis, migration, and invasion both in vitro and in vivo. Patients with higher LUCAT1 expression tend to have worse prognoses and poorer responses to chemotherapy^47,48^. Analyzing the TCGA-LIHC database, Jiao et al. found that increased LUCAT1 expression in liver cancer correlates with age, histological grade, T stage, and survival status^49^. Additionally, Zhu et al. identified elevated LUCAT1 expression in liver cancer samples through qRT-PCR, and in vitro experiments indicated a close association between high LUCAT1 expression and HCC cell proliferation and migration^50^. In our study, qRT-PCR analysis of human samples revealed that while the difference in LUCAT1 expression between cancer and adjacent tissues was not pronounced, tumor tissues exhibited higher LUCAT1 expression compared to normal liver tissues. Moreover, we found that high LUCAT1 expression was linked to higher tumor recurrence rates and histological characteristics of poorer differentiation. These factors suggest that elevated LUCAT1 expression is associated with poorer prognosis. However, in our own cohort, there was no significant difference in prognosis between high and low LUCAT1 expression, possibly due to our relatively small sample size. Further validation with a larger sample size will be necessary in the future.

Through bioinformatics analysis, we identified a bile acid and salt metabolism-related lncRNA signature associated with survival. This signature can also, to some extent, predict the response of patients to immunotherapy and chemotherapy. There are several limitations in this study. Firstly, this was a retrospective study based on data obtained from public databases. Hence, prospective cohort studies are needed to verify the accuracy of this model in the future. Secondly, these predictions were solely based on bioinformatic and comparative analyses with small scale, thus, further study with larger sample size in the real-world setting is warranted.

In conclusion, we performed a comprehensive analysis of the association between the bile acid metabolism-related lncRNA and survival of patients with HCC. A novel bile acid and bile salt metabolism-related prognostic risk signature was constructed. This signature could effectively serve as an independent prognostic biomarker and a potential predictive factor for the effectiveness of immunotherapy and chemotherapy in patients with HCC.

## Materials and methods

### Patients and datasets

We downloaded the fragments per kilobase of transcript per million mapped reads-standardized RNA-sequencing data and the corresponding clinical and prognostic data from TCGA portal (http://cancergenome.nih.gov) on January 4, 2022; the data included 374 tumor samples and 50 adjacent normal samples. Additionally, for external validation, we obtained a liver cancer dataset, GSE14520, from the Gene Expression Omnibus (GEO) database. To identify relevant gene sets related to bile acid and bile salt metabolism, we utilized the curated "HALLMARK_BILE_ACID_METABOLISM" signature from the Molecular Signatures Database (MSigDB), available at https://www.gsea-msigdb.org/gsea/msigdb. This hallmark gene set comprises 28 individual gene sets, encompassing a total of 112 genes primarily involved in the metabolism of bile acids and bile salts (Supplementary Tables S5, S6). "Hallmark" gene sets are meticulously curated to provide a more refined and consolidated representation of various biological processes or states. They offer a coherent and concise depiction of gene expression patterns associated with these processes, reducing redundancy and variability. Consequently, they serve as valuable inputs for gene set enrichment analysis, offering a more focused and streamlined alternative compared to the original gene sets. This approach enhances the interpretability and accuracy of our analysis, allowing us to investigate the specific role of bile acid metabolism-related genes in hepatocellular carcinoma (HCC) with greater precision^51^.

Fifty HCC tissues and corresponding paracancerous tissues in this study were obtained from Tianjin Third Central Hospital (Tianjin, China) after surgical resection, meanwhile, ten normal liver tissues adjacent to benign lesions (include hepatic cyst, liver cavernous hemangioma and angiomyolipoma of liver) were acquired in the same way, all samples were collected from 2015 to 2018, and were frozen and stored in – 80 °C for further analysis. All patients were followed until death or closure of data analysis on May 4, 2023.The clinical records of the patients were listed in Supplementary Table S7. All samples and relative study were approved by the Medical Ethics Committees of Tianjin Third central Hospital (Approval number: IRB2019-034-01).

### Identification and functional enrichment analysis of differentially expressed bile acid metabolism-related genes (DEGs)

DEGs were selected using the R package “limma”, with a false discovery rate < 0.05 and |log_2_ fold change (FC) > 1| as screening criteria. A total of 23 DEGs were identified for further functional enrichment analysis (Supplementary Table S1). We performed Gene Ontology (GO) and Kyoto Encyclopedia of Genes and Genomes (KEGG) analyses on these DEGs, and visualized the results with the “ggplot2” package.

### Construction of the bile acid and bile salt metabolism-related lncRNA predictive signature

Pearson's correlation analyses were performed to determine the correlation between the expression levels of lncRNAs and bile acid and bile salt metabolism-related DEGs. LncRNAs with a Pearson's correlation coefficient |r| > 0.3 and p < 0.001 were selected for further analysis. Overall, we identified 498 lncRNAs. Subsequently, we used the screened bile acid metabolism-lncRNAs to construct a prognostic risk score model for HCC. Firstly, HCC patients with incomplete follow‐up information or less than 30 days of survival were excluded. Altogether, 343 cases of HCC were randomly assigned into the training (n = 172) and validation (n = 171) sets at the ratio of 1:1 using the R project “caret” package. In the training set, 498 bile acid metabolism-related lncRNAs were analyzed by univariate Cox regression analysis using the “survival” package of R project. The results with p value > 0.05 were filtered out. Secondly, the recognized lncRNAs were further screened and confirmed via the least absolute shrinkage and selection operator (LASSO) algorithm using the R project “glmnet” package; eight LncRNAs were chosen. Thirdly, a multivariate Cox regression model was applied to those bile acid metabolism‐related lncRNAs. Finally, five bile acid metabolism‐related lncRNAs and their corresponding coefficients were identified to construct the prognostic signature in HCC. The risk score was calculated using the following formula:

$$Risk \, score=\sum \mathrm{Coef\, i}*\mathrm{Expr\, i}$$

where, Coef is the coefficient value, and Expr is the expression value of selected bile acid metabolism-related lncRNAs. This formula was used to calculate the risk score for each patient with HCC. According to the median value of the risk score, patients were divided into low- and high-risk groups. Thereafter, Kaplan–Meier analysis and receiver operating characteristic (ROC) curve analysis were used to evaluate the prognosis in these groups. In addition, multivariate Cox regression analysis was performed to examine the usefulness of the signature as an independent biomolecular indicator for the prediction of survival.

To further validate the robustness of our model, we conducted external validation using the liver cancer dataset GSE14520 from the Gene Expression Omnibus (GEO) database. However, as the GEO dataset lacked expression data for the five lncRNAs included in our model, we referred to previous literature^52,53^ and employed the Gene Set Variation Analysis (GSVA) to establish an alternative scoring system. This system was used to delineate differential gene profiles between the high-risk and low-risk groups. Utilizing the risk model based on the five lncRNAs we previously identified, we determined distinct expression features, denoted as Gene Signature A and Gene Signature B, in the low-risk and high-risk groups within the TCGA-LIHC dataset, thereby further confirming the stability of our risk model. Gene Signature A comprises genes found to be highly expressed in the high-risk group, while Gene Signature B encompasses genes highly expressed in the low-risk group. By analyzing the differences between these gene signatures, as well as their correlation with Risk Scores (RS), this method serves as an alternative risk scoring approach. Subsequently, RS scores were computed for the external GEO cohort, and the Kaplan–Meier method was applied to compare the overall survival (OS) between high RS score and low RS score groups.

### Nomogram construction

Various clinical traits, including age, sex, stage, grade, Child–Turcotte–Pugh (CTP) grade of liver function, and the risk score were incorporated to construct a prognostic nomogram that can predict the 1-, 3-, and 5-year survival of patients with HCC. Calibration curves were plotted to test whether the predicted survival rate was consistent with the actual survival rate.

### Functional enrichment analysis of the bile acid and bile salt metabolism-related lncRNA predictive signature

We carried out gene set enrichment analysis (GSEA) to investigate the mechanisms significantly correlated with our bile acid metabolism-related lncRNA predictive signature. GSEA was used to determine the pathway genes that were enriched^54^. The GSEA 4.1.0 software (http://www.broad.mit.edu/gsea/) was used for the analysis. Nominal p values < 0.05 denoted statistically significant differences.

### Estimation of tumor-infiltrating immune cells

TCGA immune cell infiltration data (e.g., CD8 + T cells, B cells, CD4 + T cells, dendritic cells, macrophages, and neutrophils) were obtained from the tumor immune estimation resource (TIMER) database (https://cistrome.shinyapps.io/timer/). Spearman correlation analysis was used to analyze the relationship between the types of immune infiltrating cells obtained through various algorithms (e.g., xCell, TIMER, CIBERSORT) and the risk score. A bubble graph was used to demonstrate the relationship. Subsequently, the low- and high-risk groups were compared for differences in immune cell infiltration. To assess the immune microenvironment in high and low-risk groups of HCC patients, the ESTIMATE algorithm^14^ which can use the RNA-seq transcriptome profiles of certain genes to quantitatively estimate the scores of stromal and immune cells in the TME was applied to calculate the stromal and immune scores in high- and low-risk groups.

### Signature and immune checkpoint blockade (ICB)

According to previous research, the expression of immune checkpoint blockade therapy-correlated genes may be associated with the responsiveness to ICB therapy^55^. We employed six key genes linked to therapy with immune checkpoint inhibitors in HCC, namely programmed death 1 (PD-1, also known as PDCD1), T-cell immunoglobulin domain and mucin domain-containing molecule-3 (TIM-3, also known as HAVCR2), cytotoxic T-lymphocyte associated antigen 4 (CTLA4), T Cell immunoreceptor with Ig and ITIM domains (TIGIT), cluster of differentiation 47 (CD47), and cluster of differentiation 276 (CD276). Spearman correlation analysis was used to explore the correlation between the above six ICB-related genes and the risk score of our model. To further explore the potential role of our lncRNA risk model in ICB treatment of patients with HCC, the expression levels of 47 immune checkpoint blockage-related genes in low- and high-risk patients were measured^56^.

### Role of the predictive signature in predicting response to clinical treatment

To assess the predictive capacity of our model regarding therapy response in HCC, we utilized the Tumor Immune Dysfunction and Exclusion (TIDE) algorithm, available at http://tide.dfci.harvard.edu/, to predict the therapeutic effectiveness of Immune Checkpoint Inhibitors (ICIs) between the high-risk and low-risk groups. Higher TIDE prediction scores indicate a greater likelihood of immune evasion, suggesting reduced responsiveness to ICI treatment. In a further exploration of our bile acid and salt metabolism related lncRNA risk model's predictive performance in the context of tumor immunotherapy, we employed the Risk Score (RS) as an alternative to the risk score to evaluate response in the anti-PDL1 cohort (IMvigor 210). Additionally, we leveraged the "pRRophetic" package within the R project to infer the sensitivity of different risk groups to various chemotherapeutic and targeted drugs. We calculated the half-maximal inhibitory concentration (IC_50_) for commonly used clinical HCC treatment drugs. The estimated IC_50_ values for the low-risk and high-risk groups were compared using the Wilcoxon rank-sum test.

### RNA extraction and real-time quantitative reverse transcription polymerase chain reaction (qRT-PCR)

Total RNA was extracted from liver tissue using Trizol reagent (Takara, Dalian, China) following the manufacturer's instructions and referencing prior literature^57^. RNA concentration and quality were assessed using a NanoDrop ND-2000 spectrophotometer (Life Technologies, Grand Island, NY, USA). RNA concentration and quality were assessed using a NanoDrop ND-2000 spectrophotometer (Life Technologies, Grand Island, NY, USA). First-strand cDNA synthesis was performed using GoScript™ Reverse Transcriptase (Promega (Beijing) Biotech Co. Ltd). SYBR Green real-time PCR was conducted using TaKaRa Ex Taq® Hot Start Version (Takara, Beijing, China). Primer information can be found in Supplementary Table S8. The amplification conditions included an initial denaturation step at 95 °C for 3 min, followed by 40 cycles of denaturation at 94 °C for 45 s, annealing at 60 °C for 1 min. All real-time PCR experiments were conducted using the QuantStudio™5 Real-Time Fluorescence Quantitative PCR System (Thermo Fisher SCIENTIFIC). Each experiment was repeated three times for accuracy. To ensure reliable quantification, GAPDH mRNA expression levels were used as a reference for normalization. The amplification efficiencies for each LncRNA and GAPDH were consistent. Quantitative analysis was performed using the 2^−ΔΔCT^ method.

### Statistical analysis

All statistical analyses were performed with the R software (Version 4.0.5; R Foundation). GSEA (http://www.broadinstitute.org/gsea/index.jsp) was used to distinguish between two sets of functional annotations. All p values are two-side, and values < 0.05 denoted statistically significant differences.

### Ethics approval and consent to participate

This study was approved by the Ethics Committee of Tianjin Third central Hospital (Approval number: IRB2019-034-01). All procedures involving human participants were in accordance with the ethical standards of national committee and the Helsinki declaration. Informed consent was obtained from all participants and/or their legal guardians.

### Supplementary Information

Supplementary Information.

## Data Availability

Collections of data from this study are available in public databases. The names of the repository/repositories are included in the article/Supplementary Material. Further inquiries can be directed to the corresponding author.
