# Supplementary material for: Identification of a bile acid and bile salt metabolism-related lncRNA signature for predicting prognosis and treatment response in hepatocellular carcinoma
Source: Sci Rep. 2023 Nov 9;13:19512. doi: 10.1038/s41598-023-46805-6 (PMC10636107; doi:10.1038/s41598-023-46805-6)
Supplement: Supplementary file 1 — Supplementary Information. [file 41598_2023_46805_MOESM1_ESM.pdf]

Identification of a Bile Acid and Bile Salt Metabolism-related lncRNA Signature for Predicting Prognosis and Treatment Response in Hepatocellular Carcinoma

Hao Cui, Jia Lian，Baiguo Xu, Zhenjun Yu, Huiling Xiang，Jingxiang Shi, Yingtang Gao, Tao Han

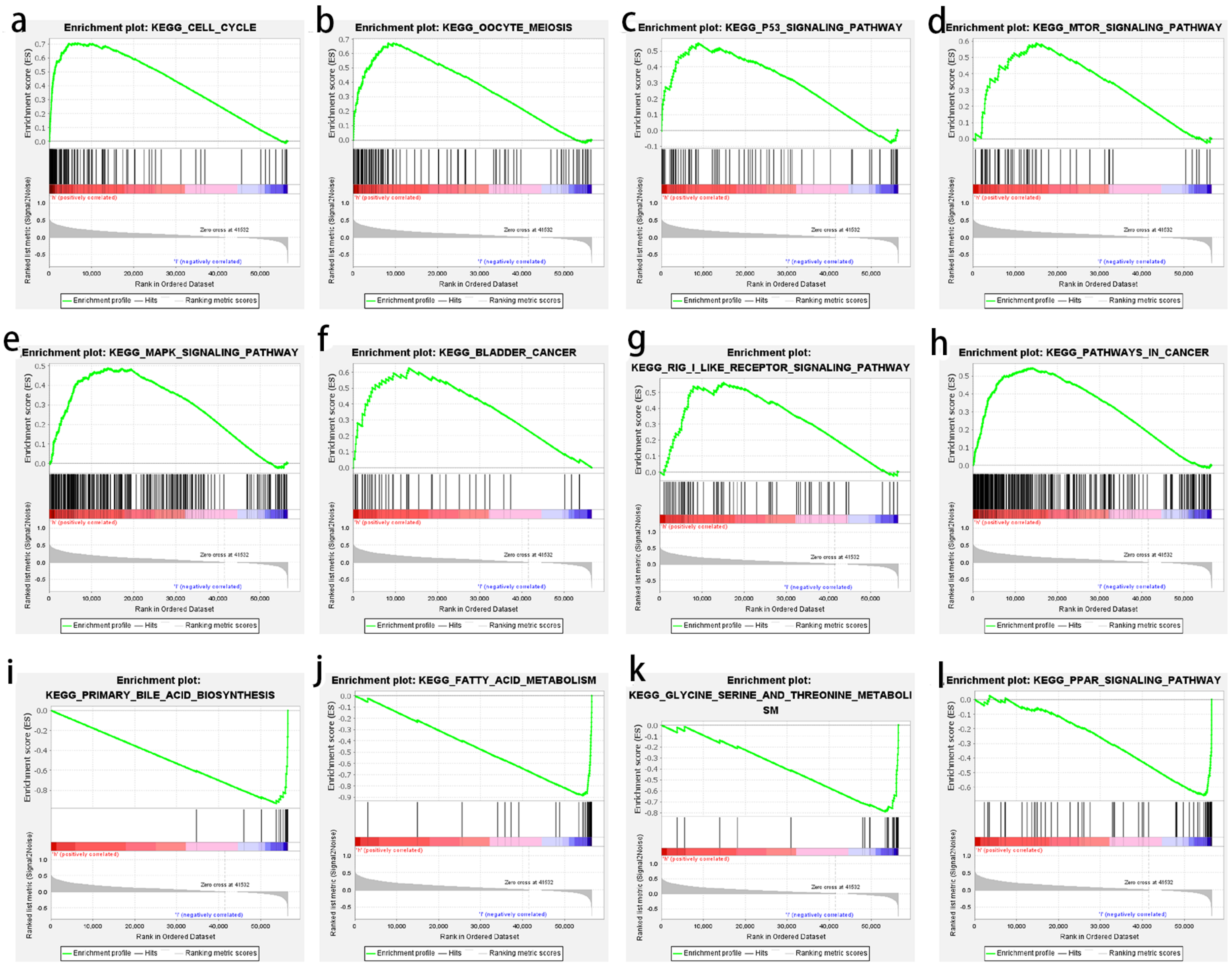

- Figure S1 Activated pathways involved in the bile acid metabolism-related gene signature, as shown by GSEA. The following were activated in the high-risk group: (a) Cell cycle; (b) Oocyte meiosis; (c) p53 signaling pathway; (d) mTOR signaling pathway; (e) MAPK signaling pathway; (f) Bladder cancer; (g) RIG-I like receptor signaling pathway; and (h) pathway in Cancer. The following were activated in the low-risk group: (i) Primary bile acid biosynthesis; (j) fatty acid metabolism; (k) Glycine serine and threonine metabolism; and (l) PPAR signaling pathway.

- GSEA, gene set enrichment analysis; KEGG, Kyoto Encyclopedia of Genes and Genomes; MAPK, mitogen-activated protein kinase; mTOR, mammalian target of rapamycin; PPAR, peroxisome proliferator-activated receptor.

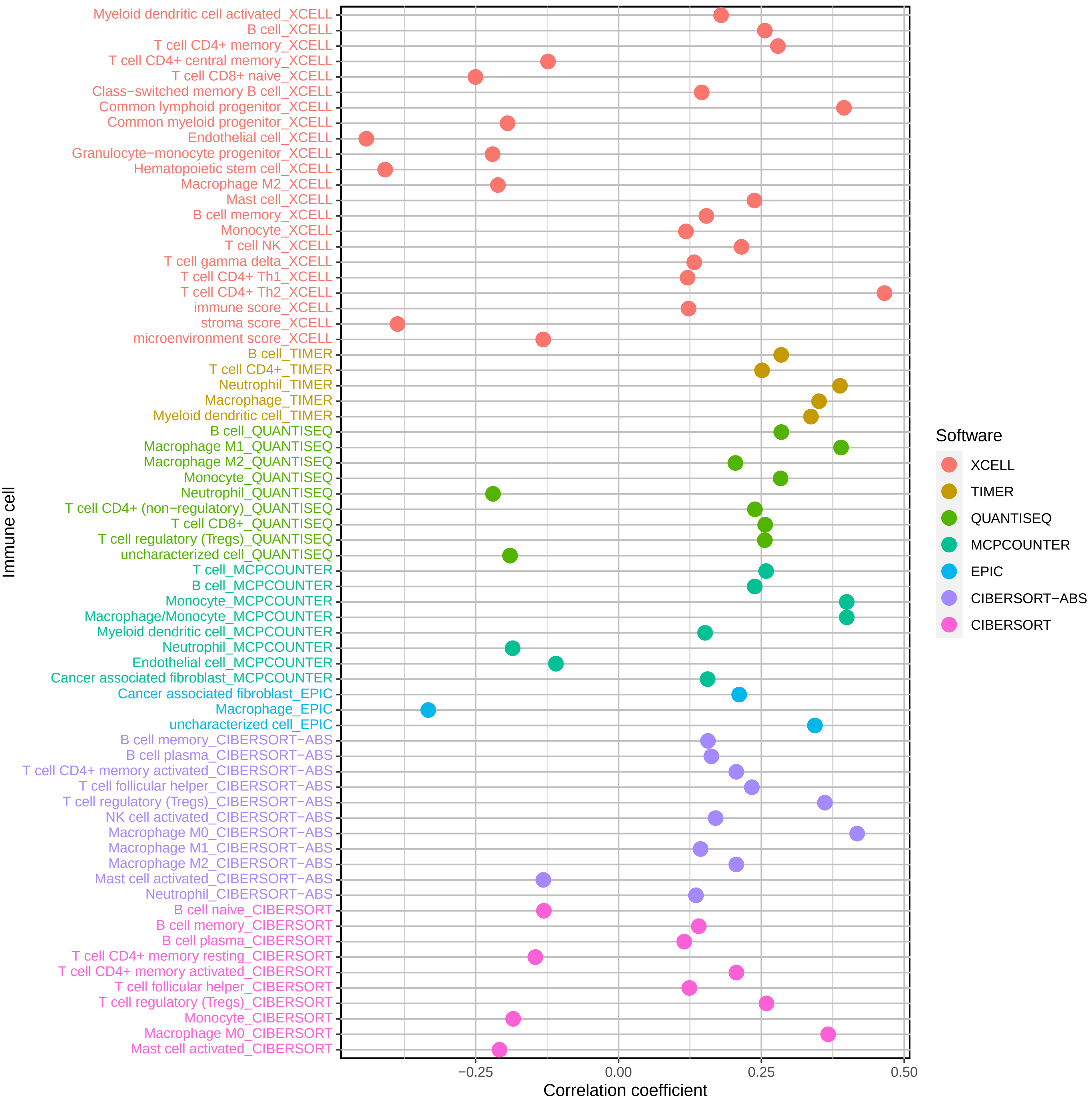
Myeloid dendritic cell activated_XCELL
B cell_XCELL
T cell CD4+ memory_XCELL
T cell CD4+ central memory_XCELL
T cell CD8+ naive_XCELL
Class−switched memory B cell_XCELL
Common lymphoid progenitor_XCELL
Common myeloid progenitor_XCELL
Endothelial cell_XCELL
Granulocyte−monocyte progenitor_XCELL
Hematopoietic stem cell_XCELL
Macrophage M2_XCELL
Mast cell_XCELL
B cell memory_XCELL
Monocyte_XCELL
T cell NK_XCELL
T cell gamma delta_XCELL
T cell CD4+ Th1_XCELL
T cell CD4+ Th2_XCELL
immune score_XCELL
stroma score_XCELL
microenvironment score_XCELL
B cell_TIMER
T cell CD4+_TIMER
Neutrophil_TIMER
Macrophage_TIMER
Myeloid dendritic cell_TIMER
B cell_QUANTISEQ
Macrophage M1_QUANTISEQ
Macrophage M2_QUANTISEQ
Monocyte_QUANTISEQ
Neutrophil_QUANTISEQ
T cell CD4+ (non−regulatory)_QUANTISEQ
T cell CD8+_QUANTISEQ
T cell regulatory (Tregs)_QUANTISEQ
uncharacterized cell_QUANTISEQ
T cell_MCPCOUNTER
B cell_MCPCOUNTER
Monocyte_MCPCOUNTER
Macrophage/Monocyte_MCPCOUNTER
Myeloid dendritic cell_MCPCOUNTER
Neutrophil_MCPCOUNTER
Endothelial cell_MCPCOUNTER
Cancer associated fibroblast_MCPCOUNTER
Cancer associated fibroblast_EPIC
Macrophage_EPIC
uncharacterized cell_EPIC
B cell memory_CIBERSORT−ABS
B cell plasma_CIBERSORT−ABS
T cell CD4+ memory activated_CIBERSORT−ABS
T cell follicular helper_CIBERSORT−ABS
T cell regulatory (Tregs)_CIBERSORT−ABS
NK cell activated_CIBERSORT−ABS
Macrophage M0_CIBERSORT−ABS
Macrophage M1_CIBERSORT−ABS
Macrophage M2_CIBERSORT−ABS
Mast cell activated_CIBERSORT−ABS
Neutrophil_CIBERSORT−ABS
B cell naive_CIBERSORT
B cell memory_CIBERSORT
B cell plasma_CIBERSORT
T cell CD4+ memory resting_CIBERSORT
T cell CD4+ memory activated_CIBERSORT
T cell follicular helper_CIBERSORT
T cell regulatory (Tregs)_CIBERSORT
Monocyte_CIBERSORT
Macrophage M0_CIBERSORT
Mast cell activated_CIBERSORT
Immune cell
−0.25
0.00
0.25
0.50
Correlation coefficient
Software
XCELL
TIMER
QUANTISEQ
MCPCOUNTER
EPIC
CIBERSORT−ABS
CIBERSORT

Figure S2 A detailed Spearman correlation analysis was performed using different algorithms; a lollipop shape to exhibit the results. According to the findings, most immune cells had a positive correlation with the risk score.
TIMER, tumor immune estimation resource.

gene	conMean	treatMean	logFC	pValue	fdr
ABCA2	2.94961202	5.984880364	1.020797208	1.32232E-09	3.50237E-09
ABCA3	0.298205757	1.446240247	2.277927215	1.67473E-11	5.47079E-11
ABCA4	0.088847755	0.321946178	1.857412289	3.28671E-10	9.2028E-10
ABCD1	2.7213672	8.418065719	1.629157137	5.04799E-21	9.89407E-20
ACSL1	181.0926394	77.88915995	-1.217233445	8.80493E-18	1.0786E-16
AKR1D1	49.2878328	19.92278709	-1.30681206	8.76636E-16	6.60849E-15
ALDH8A1	66.4092684	26.05747895	-1.349687095	7.98257E-25	3.91146E-23
BBOX1	12.28859707	5.500818653	-1.159601972	4.38641E-13	2.00788E-12
CYP39A1	16.71967308	4.517599331	-1.887918411	9.95221E-23	3.25105E-21
CYP7A1	5.019147193	28.06688766	2.483354931	0.000351665	0.000514376
DIO2	0.015357284	0.479832795	4.965536753	2.15414E-08	5.02634E-08
EFHC1	0.266598837	0.738479607	1.469887595	8.34638E-18	1.0786E-16
FADS1	4.648333576	12.25443472	1.398518428	4.00717E-06	7.40949E-06
FADS2	9.642111654	20.05759983	1.056727937	0.011186336	0.014237154
GNPAT	8.1486725	23.37881262	1.520564705	1.97747E-27	1.93792E-25
KLF1	0.025191275	0.061764947	1.293864148	0.03592091	0.042412641
LIPE	0.195530221	0.598194954	1.613224132	0.000248678	0.000369249
NPC1	1.411265224	3.746963768	1.408732883	1.76835E-20	2.8883E-19
PEX6	8.47706206	18.40213118	1.118236603	5.15587E-15	3.3685E-14
PFKM	0.866943006	2.25856606	1.381398054	3.06664E-06	6.13328E-06
SLC27A5	99.2047594	49.3965942	-1.005997762	4.3439E-14	2.36501E-13
SLC29A1	13.72519202	36.7948889	1.422679045	1.18204E-13	6.09684E-13
SLC35B2	12.93416866	30.45116345	1.235310022	4.29762E-22	1.05292E-20

Supplementary Table S1 - Differentially Expressed Bile Acid and Bile Salt Metabolism-related Genes

A total of 23 differentially expressed genes (DEGs) were identified using a false discovery

NAME	SIZE	ES	NES	NOM p-val	FDR q-val	FWER p-val
KEGG_OOCYTE_MEIOSIS	112	0.67141706	2.1545446	0	0.001333333	0.001
KEGG_SPLICEOSOME	127	0.7897066	2.0736427	0	0.010688174	0.004
KEGG_RNA_DEGRADATION	59	0.72558856	2.0691977	0	0.007125449	0.004
KEGG_PYRIMIDINE_METABOLISM	97	0.6221806	2.057625	0	0.00567742	0.004
KEGG_UBIQUITIN_MEDIATED_PROTEOLYSIS	134	0.67652166	2.0563598	0	0.004541936	0.004
KEGG_PURINE_METABOLISM	158	0.5621436	2.0378401	0	0.004526996	0.006
KEGG_CELL_CYCLE	124	0.7065776	2.0233965	0	0.004663211	0.009
KEGG_NOD_LIKE_RECEPTOR_SIGNALING_PATHWAY	62	0.6750451	1.9968933	0	0.004358087	0.01
KEGG_ENDOCYTOSIS	181	0.6005543	1.9891582	0	0.00481083	0.017
KEGG_AMINOACYL_TRNA_BIOSYNTHESIS	41	0.72851515	1.961551	0	0.006062158	0.027
KEGG_VASOPRESSIN_REGULATED_WATER_REABSORPTION	44	0.6786817	1.9492435	0	0.006573737	0.033
KEGG_BASE_EXCISION_REPAIR	35	0.71150553	1.9401464	0	0.006833883	0.04
KEGG_REGULATION_OF_ACTIN_CYTOSKELETON	213	0.5713703	1.9093891	0	0.009998886	0.059
KEGG_HOMOLOGOUS_RECOMBINATION	28	0.7508321	1.8999968	0	0.00961725	0.065
KEGG_FC_GAMMA_R_MEDIATED_PHAGOCYTOSIS	96	0.64598376	1.8913475	0	0.010091268	0.071
KEGG_MISMATCH_REPAIR	23	0.78235805	1.8879602	0	0.009943951	0.073
KEGG_BLADDER_CANCER	42	0.6252679	1.8863624	0	0.009595111	0.073
KEGG_PATHWAYS_IN_CANCER	325	0.54452807	1.881648	0	0.009439097	0.073
KEGG_EPITHELIAL_CELL_SIGNALING_IN_HELICOBACTER_PYLORI_INFECTION	68	0.6012803	1.8811853	0	0.009065375	0.074
KEGG_NEUROTROPHIN_SIGNALING_PATHWAY	126	0.6028199	1.8689362	0	0.010770182	0.09
KEGG_PROGESTERONE_MEDIATED_OOCYTE_MATURATION	85	0.59748524	1.8548752	0	0.010736099	0.102
KEGG_BASAL_TRANSCRIPTION_FACTORS	35	0.66157943	1.8517637	0	0.010904925	0.109
KEGG_NUCLEOTIDE_EXCISION_REPAIR	44	0.66642904	1.8474228	0	0.010759453	0.114
KEGG_VIBRIO_CHOLERAE_INFECTION	54	0.596846	1.839141	0	0.011603302	0.126
KEGG_P53_SIGNALING_PATHWAY	68	0.54998815	1.8305464	0	0.012617771	0.136
KEGG_RIG_I_LIKE_RECEPTOR_SIGNALING_PATHWAY	71	0.5608659	1.8112013	0	0.015924782	0.157
KEGG_MTOR_SIGNALING_PATHWAY	52	0.5874999	1.7691246	0	0.021248706	0.223
KEGG_MAPK_SIGNALING_PATHWAY	267	0.488321	1.7279301	0	0.029728884	0.303
KEGG_CYTOSOLIC_DNA_SENSING_PATHWAY	55	0.59147507	1.86384	0.001949318	0.010060634	0.094
KEGG_RNA_POLYMERASE	28	0.69869787	1.8664647	0.001956947	0.010539388	0.09
KEGG_OTHER_GLYCAN_DEGRADATION	16	0.7137133	1.8556408	0.002024292	0.011109433	0.102
KEGG_PATHOGENIC_ESCHERICHIA_COLI_INFECTION	56	0.65148556	1.9038407	0.002057613	0.009611081	0.062
KEGG_PANCREATIC_CANCER	70	0.62416	1.8513529	0.002074689	0.010664255	0.11
KEGG_NOTCH_SIGNALING_PATHWAY	47	0.6365257	1.8362604	0.00209205	0.011906378	0.129
KEGG_DNA_REPLICATION	36	0.7755696	1.8660856	0.003861004	0.010100247	0.09
KEGG_FC_EPSILON_RI_SIGNALING_PATHWAY	79	0.5325566	1.7267871	0.003960396	0.028590823	0.303
KEGG_SNARE_INTERACTIONS_IN_VESICULAR_TRANSPORT	38	0.599617	1.7270845	0.004081633	0.029178157	0.303
KEGG_GLYCOSPHINGOLIPID_BIOSYNTHESIS_LACTO_AND_NEOLACTO_SERIES	26	0.57432884	1.7139038	0.004081633	0.029524716	0.33
KEGG_ADHERENS_JUNCTION	73	0.5953842	1.7887859	0.00409836	0.0188723	0.191
KEGG_LYSOSOME	121	0.5878188	1.9306786	0.004132231	0.007174629	0.043
KEGG_NON_SMALL_CELL_LUNG_CANCER	54	0.5716724	1.7430662	0.004132231	0.027405083	0.272
KEGG_WNT_SIGNALING_PATHWAY	150	0.5229735	1.7439542	0.004149378	0.027692	0.268
KEGG_SMALL_CELL_LUNG_CANCER	84	0.5865402	1.8001357	0.004192872	0.017416595	0.176
KEGG_CHRONIC_MYELOID_LEUKEMIA	73	0.6005306	1.7973073	0.00422833	0.017501187	0.181
KEGG_AMINO_SUGAR_AND_NUCLEOTIDE_SUGAR_METABOLISM	43	0.53667283	1.6954256	0.006122449	0.031639352	0.37
KEGG_COLORECTAL_CANCER	62	0.5982555	1.7751021	0.00631579	0.020350669	0.211
KEGG_LEISHMANIA_INFECTION	70	0.653148	1.7765945	0.008064516	0.020594409	0.209
KEGG_APOPTOSIS	87	0.5523597	1.7280699	0.008264462	0.030389527	0.303
KEGG_HEDGEHOG_SIGNALING_PATHWAY	56	0.55159974	1.7086627	0.008474576	0.029989589	0.334
KEGG_TOLL_LIKE_RECEPTOR_SIGNALING_PATHWAY	102	0.541993	1.7055405	0.008658009	0.029951459	0.348
KEGG_VEGF_SIGNALING_PATHWAY	76	0.5109987	1.7049482	0.009940358	0.029562194	0.348
KEGG_INSULIN_SIGNALING_PATHWAY	136	0.48681387	1.718907	0.010351967	0.029555123	0.32
KEGG_ERBB_SIGNALING_PATHWAY	87	0.5402168	1.7076762	0.010438413	0.0297506	0.338
KEGG_TIGHT_JUNCTION	131	0.46547952	1.604218	0.010548524	0.052345008	0.556
KEGG_INOSITOL_PHOSPHATE_METABOLISM	54	0.57581866	1.6655653	0.010660981	0.037730128	0.427
KEGG_N_GLYCAN_BIOSYNTHESIS	46	0.5886489	1.722841	0.010752688	0.02871586	0.308
KEGG_AMYOTROPHIC_LATERAL_SCLEROSIS_ALS	53	0.45941672	1.6004992	0.012345679	0.053710215	0.57
KEGG_AXON_GUIDANCE	129	0.5522184	1.7862315	0.012422361	0.01926421	0.195
KEGG_GAP_JUNCTION	90	0.5279394	1.6583672	0.012422361	0.03960051	0.44
KEGG_RENAL_CELL_CARCINOMA	70	0.57855076	1.7169952	0.012875536	0.029268546	0.324
KEGG_LONG_TERM_POTENTIATION	70	0.511363	1.6764461	0.014028057	0.03582466	0.405
KEGG_T_CELL_RECEPTOR_SIGNALING_PATHWAY	108	0.58638775	1.7326608	0.014314928	0.029659914	0.293
KEGG_GNRH_SIGNALING_PATHWAY	101	0.5066077	1.6758463	0.018218623	0.035471685	0.407
KEGG_GLYCOSPHINGOLIPID_BIOSYNTHESIS_GANGLIO_SERIES	15	0.6096098	1.6251249	0.019569471	0.04840774	0.51
KEGG_PROTEASOME	46	0.6643607	1.676694	0.01980198	0.036330584	0.404
KEGG_GLYCEROPHOSPHOLIPID_METABOLISM	76	0.42809743	1.5154908	0.02020202	0.07589294	0.727
KEGG_REGULATION_OF_AUTOPHAGY	35	0.5169061	1.6144775	0.020242915	0.050759584	0.537
KEGG_PHOSPHATIDYLINOSITOL_SIGNALING_SYSTEM	76	0.55059373	1.6409864	0.020876827	0.04458351	0.467
KEGG_MELANOGENESIS	101	0.47756094	1.6091609	0.021141648	0.051200744	0.549
KEGG_B_CELL_RECEPTOR_SIGNALING_PATHWAY	75	0.55206907	1.6152155	0.022680413	0.051267847	0.534
KEGG_ACUTE_MYELOID_LEUKEMIA	57	0.5310453	1.5891665	0.02771855	0.05624182	0.592
KEGG_MELANOMA	71	0.45447257	1.5059332	0.029535865	0.077555425	0.741
KEGG_THYROID_CANCER	29	0.55053043	1.5745571	0.029940119	0.06029894	0.624
KEGG_GLIOMA	65	0.49694616	1.5819875	0.031185031	0.058004454	0.608
KEGG_HUNTINGTONS_DISEASE	180	0.49166292	1.6409272	0.03250478	0.043886892	0.467
KEGG_PROSTATE_CANCER	89	0.49867132	1.5991836	0.03411514	0.052927293	0.573
KEGG_SELENOAMINO_ACID_METABOLISM	26	0.5349112	1.5730563	0.035714287	0.06036694	0.628
KEGG_JAK_STAT_SIGNALING_PATHWAY	155	0.46266073	1.5494349	0.037848607	0.06669442	0.666
KEGG_LONG_TERM_DEPRESSION	70	0.4614431	1.5249714	0.037848607	0.07432658	0.704
KEGG_SPHINGOLIPID_METABOLISM	39	0.51040477	1.5426228	0.03821656	0.068255015	0.682
KEGG_VIRAL_MYOCARDITIS	68	0.55360866	1.6342583	0.039175257	0.045844417	0.487
KEGG_DORSO_VENTRAL_AXIS_FORMATION	24	0.58615553	1.5668943	0.039337475	0.06213631	0.637
KEGG_GLYCOSYLPHOSPHATIDYLINOSITOL_GPI_ANCHOR_BIOSYNTHESIS	25	0.5852584	1.6136312	0.044487428	0.050275665	0.539
KEGG_GLYCOSAMINOGLYCAN_BIOSYNTHESIS_CHONDROITIN_SULFATE	22	0.5866452	1.5499221	0.045454547	0.067360274	0.665
KEGG_PRION_DISEASES	35	0.49906236	1.5447385	0.045908183	0.0676986	0.673
KEGG_ENDOMETRIAL_CANCER	52	0.5201984	1.5632073	0.047916666	0.062920205	0.647
KEGG_CHEMOKINE_SIGNALING_PATHWAY	188	0.488172	1.5826169	0.0499002	0.058545254	0.606

Supplementary Table S2 Significantly enriched pathways in the high-risk group by GSEA analysis

87 pathways significantly enriched in the high-risk group. ES, Enrichment Score. NES, Normalized Enrichment Score. NOM p-val, nominal P value. FDR q-val, false discovery rate q-val. FWER p-val： familywise-error rate p-val.

NAME	SIZE	ES	NES	NOM p-val	FDR q-val	FWER p-val
KEGG_FATTY_ACID_METABOLISM	42	-0.88597286	-2.1997712	0	0	0
KEGG_DRUG_METABOLISM_CYTOCHROME_P450	71	-0.71342415	-2.116025	0	0	0
KEGG_PPAR_SIGNALING_PATHWAY	69	-0.6573673	-2.098458	0	0	0
KEGG_GLYCINE_SERINE_AND_THREONINE_METABOLISM	31	-0.7917958	-2.068537	0	0.00053347	0.004
KEGG_TRYPTOPHAN_METABOLISM	40	-0.70766264	-2.0643997	0	0.00054659	0.006
KEGG_PRIMARY_BILE_ACID_BIOSYNTHESIS	16	-0.93063205	-2.0562623	0	0.000455491	0.006
KEGG_VALINE_LEUCINE_AND_ISOLEUCINE_DEGRADATION	44	-0.8317151	-2.0489957	0	0.000437219	0.007
KEGG_COMPLEMENT_AND_COAGULATION_CASCADES	69	-0.74491847	-2.0441453	0	0.000382567	0.007
KEGG_RETINOL_METABOLISM	64	-0.6866099	-2.0365908	0	0.000450937	0.008
KEGG_PEROXISOME	78	-0.63863546	-1.9361193	0	0.002202441	0.038
KEGG_BUTANOATE_METABOLISM	34	-0.7208831	-1.9449444	0.001926782	0.002142798	0.032
KEGG_PROPANOATE_METABOLISM	33	-0.7267457	-1.884031	0.001934236	0.004316084	0.072
KEGG_LINOLEIC_ACID_METABOLISM	29	-0.6186314	-1.9072527	0.002178649	0.003208187	0.055
KEGG_ARGININE_AND_PROLINE_METABOLISM	54	-0.5323407	-1.7759609	0.006048387	0.014386518	0.211
KEGG_METABOLISM_OF_XENOBIOTICS_BY_CYTOCHROME_P450	69	-0.5806537	-1.7613549	0.007905139	0.015008633	0.237
KEGG_TYROSINE_METABOLISM	42	-0.5410026	-1.7237866	0.008230452	0.019953776	0.303
KEGG_HISTIDINE_METABOLISM	29	-0.5674721	-1.7246519	0.010460251	0.021008674	0.302
KEGG_BETA_ALANINE_METABOLISM	22	-0.69382775	-1.7754564	0.01171875	0.013491479	0.211
KEGG_ALANINE_ASPARTATE_AND_GLUTAMATE_METABOLISM	32	-0.49199042	-1.5733231	0.024291499	0.06564061	0.613
KEGG_PHENYLALANINE_METABOLISM	18	-0.5730748	-1.5823413	0.028629856	0.06449389	0.6
KEGG_ARACHIDONIC_ACID_METABOLISM	58	-0.40364382	-1.454661	0.033195022	0.1208959	0.827
KEGG_STEROID_HORMONE_BIOSYNTHESIS	55	-0.49234486	-1.4801935	0.0562249	0.11455649	0.781
KEGG_DRUG_METABOLISM_OTHER_ENZYMES	51	-0.462523	-1.469079	0.064150944	0.116467446	0.801
KEGG_NITROGEN_METABOLISM	23	-0.49357456	-1.4143913	0.07368421	0.1382117	0.881
KEGG_PYRUVATE_METABOLISM	40	-0.45760593	-1.4201078	0.099236645	0.1396538	0.875
KEGG_GLYOXYLATE_AND_DICARBOXYLATE_METABOLISM	16	-0.5134472	-1.3960748	0.11201629	0.1460449	0.901
KEGG_STARCH_AND_SUCROSE_METABOLISM	51	-0.39353505	-1.2376574	0.17890772	0.260282	0.991
KEGG_GLYCOLYSIS_GLUCONEOGENESIS	61	-0.35235202	-1.1942024	0.19961612	0.27605245	0.995
KEGG_BIOSYNTHESIS_OF_UNSATURATED_FATTY_ACIDS	22	-0.47062808	-1.2497811	0.20669292	0.25678605	0.987
KEGG_ASCORBATE_AND_ALDARATE_METABOLISM	25	-0.5086474	-1.2889407	0.20676692	0.2284641	0.971
KEGG_PROXIMAL_TUBULE_BICARBONATE_RECLAMATION	23	-0.41509473	-1.2058247	0.21637426	0.2732007	0.995
KEGG_CITRATE_CYCLE_TCA_CYCLE	31	-0.472004	-1.2260344	0.2854369	0.2622571	0.993
KEGG_ABC_TRANSPORTERS	44	-0.34043562	-1.1084503	0.2935606	0.34543234	0.998
KEGG_LYSINE_DEGRADATION	44	-0.3732087	-1.116545	0.35882354	0.34732783	0.998
KEGG_PENTOSE_AND_GLUCURONATE_INTERCONVERSIONS	28	-0.33601344	-0.89689434	0.5785441	0.59140646	1
KEGG_TERPENOID_BACKBONE_BIOSYNTHESIS	15	-0.3113347	-0.7542834	0.7322835	0.77676046	1
KEGG_ALPHA_LINOLENIC_ACID_METABOLISM	19	-0.24341546	-0.7219852	0.8705637	0.8021991	1
KEGG_STEROID_BIOSYNTHESIS	17	-0.2579147	-0.58267474	0.91078836	0.9340627	1

Supplementary Table S3 Significantly enriched pathways in the low-risk group by GSEA analysis

ES, Enrichment Score. NES, Normalized Enrichment Score. NOM p-val, nominal P value. FDR q-val, false discovery rate q-val. FWER p-val： familywise-error rate p-val.

Gene Name
SIRPA
CD200
TNFRSF14
NRP1
LAIR1
TNFSF4
CD244
LAG3
ICOS
CD40LG
CTLA4
CD48
CD28
CD200R1
HAVCR2
ADORA2A
CD276
KIR3DL1
CD80
PDCD1
LGALS9
CD47
TNFSF14
IDO2
ICOSLG
TMIGD2
VTCN1
IDO1
PDCD1LG2
HHLA2
TNFSF18
BTNL2
CD70
TNFSF9
TNFRSF8
CD27
TNFRSF25
VSIR
TNFRSF4
CD40
TNFRSF18
TNFSF15
TIGIT
CD274
CD86
CD44
TNFRSF9

Supplementary Table S4 47 Immune checkpoint genes

Gene Names of "HALLMARK_BILE_ACID_METABOLISM"

ABCA1	HSD3B7
ABCA2	IDH1
ABCA3	IDH2
ABCA4	IDI1
ABCA5	ISOC1
ABCA6	KLF1
ABCA8	LCK
ABCA9	LIPE
ABCD1	LONP2
ABCD2	MLYCD
ABCD3	NEDD4
ABCG4	NPC1
ABCG8	NR0B2
ACSL1	NR1H4
ACSL5	NR1I2
AGXT	NR3C2
AKR1D1	NUDT12
ALDH1A1	OPTN
ALDH8A1	PAOX
ALDH9A1	PECR
AMACR	PEX1
APOA1	PEX11A
AQP9	PEX11G
AR	PEX12
ATXN1	PEX13
BBOX1	PEX16
BCAR3	PEX19
BMP6	PEX26
CAT	PEX6
CH25H	PEX7
CROT	PFKM
CYP27A1	PHYH
CYP39A1	PIPOX
CYP46A1	PNPLA8
CYP7A1	PRDX5
CYP7B1	PXMP2
CYP8B1	RBP1
DHCR24	RETSAT
DIO1	RXRA
DIO2	RXRG
EFHC1	SCP2
EPHX2	SERPINA6
FADS1	SLC22A18
FADS2	SLC23A1
FDXR	SLC23A2
GC	SLC27A2
GCLM	SLC27A5
GNMT	SLC29A1
GNPAT	SLC35B2
GSTK1	SLCO1A2
HACL1	SOAT2
HAO1	SOD1
HSD17B11	SULT1B1
HSD17B4	SULT2B1
HSD17B6	TFCP2L1
HSD3B1	TTR

Supplementary Table S5 Bile acid metabolism-related gene sets

112 bile acid metabolism-related genes were acquired from the “HALLMARK_BILE_ACID_METABOLISM” signature of the Molecular Signatures Database(http://www.gsea-msigdb.org/gsea/msigdb/index.jsp)

Founder gene sets for the hallmark gene set :HALLMARK_BILE_ACID_METABOLISM
chr11p
chr15q
GOBP_BILE_ACID_METABOLIC_PROCESS
GOBP_HORMONE_METABOLIC_PROCESS
GOBP_PEROXISOME_ORGANIZATION
GOBP_RESPONSE_TO_DRUG
GOBP_STEROID_BIOSYNTHETIC_PROCESS
GOBP_STEROID_METABOLIC_PROCESS
GOCC_MICROBODY
GOCC_MICROBODY_MEMBRANE
GOMF_NUCLEOBASE_CONTAINING_COMPOUND_TRANSMEMBRANE_TRANSPORTER_ACTIVITY
GOMF_PROTEIN_C_TERMINUS_BINDING
KEGG_ABC_TRANSPORTERS
KEGG_PEROXISOME
KEGG_PRIMARY_BILE_ACID_BIOSYNTHESIS
MICROBODY_PART
MODULE_404
PEROXISOMAL_MEMBRANE
PEROXISOMAL_PART
PEROXISOME
REACTOME_ABC_FAMILY_PROTEINS_MEDIATED_TRANSPORT
REACTOME_ABC_TRANSPORTERS_IN_LIPID_HOMEOSTASIS
REACTOME_ALPHA_LINOLENIC_ACID_ALA_METABOLISM
REACTOME_BILE_ACID_AND_BILE_SALT_METABOLISM
REACTOME_PEROXISOMAL_LIPID_METABOLISM
REACTOME_SYNTHESIS_OF_BILE_ACIDS_AND_BILE_SALTS
REACTOME_SYNTHESIS_OF_BILE_ACIDS_AND_BILE_SALTS_VIA_24_HYDROXYCHOLESTEROL
REACTOME_SYNTHESIS_OF_BILE_ACIDS_AND_BILE_SALTS_VIA_7ALPHA_HYDROXYCHOLESTEROL

Supplementary Table S6 28 founder gene sets for the hallmark gene set：
HALLMARK_BILE_ACID_METABOLISM

ID	Sex	Age	LUCAT-Expression	AL031985.3-Expression	OS	OS time(Days)	Recurrence	RFS(Days)	T	N	M	Size(cm)	ALT	AST	TBIL	ALB	ALP	GGT	INR	Child-pugh Grade	Differentiation
1126	male	21	low-expression	low-expression	Alive	2778	No	2778	3	0	0	9	>1ULN	Normal	Normal	Normal	Normal	>3ULN	1.04	A	well
1127	male	59	no significant change	no significant change	Alive	2778	Yes	623	3	0	0	8.2	>3ULN	>3ULN	>1ULN	Normal	>1ULN	>3ULN	1.17	A	well
1130	female	58	low-expression	low-expression	Death	1032	Yes	656	2	0	0	7	Normal	Normal	Normal	Normal	Normal	Normal	0.94	A	moderately or poorly
1146	male	60	low-expression	low-expression	Alive	2752	No	2752	3	0	0	11	Normal	Normal	>1ULN	Normal	Normal	Normal	1.10	A	well
1153	male	44	no significant change	no significant change	Alive	2737	Not available	Not available	2	0	0	4	>1ULN	>1ULN	Normal	Normal	Normal	Normal	1.10	A	moderately or poorly
1167	female	83	high-expression	no significant change	Death	1379	Yes	931	2	0	0	4.7	Normal	Normal	Normal	Normal	Normal	Normal	1.02	A	well
1172	male	56	low-expression	low-expression	Alive	1996	No	1996	4	1	0	10	Normal	Normal	Normal	Normal	Normal	>1ULN	1.00	A	moderately or poorly
1174	male	56	low-expression	no significant change	Death	426	Yes	61	3	1	0	13.2	>1ULN	>1ULN	Normal	Normal	>1ULN	>3ULN	0.90	A	well
1182	female	75	no significant change	no significant change	Death	90	Yes	65	4	1	0	21	Normal	>1ULN	Normal	Normal	Normal	>1ULN	1.20	A	moderately or poorly
1202	male	62	no significant change	no significant change	Alive	2688	Yes	236	2	0	0	3	Normal	Normal	>1ULN	Normal	Normal	Normal	1.10	A	well
1203	male	62	high-expression	high-expression	Alive	2695	Yes	1236	3	0	0	7	Normal	Normal	Normal	Normal	Normal	Normal	1.00	A	well
1217	female	56	low-expression	low-expression	Alive	3285	No	3285	2	0	0	5.6	Normal	Normal	Normal	Normal	Normal	Normal	0.96	A	well
1220	male	61	no significant change	high-expression	Death	2233	Yes	285	2	0	0	7	Normal	Normal	Normal	Normal	Normal	>1ULN	1.10	A	moderately or poorly
1225	male	59	low-expression	no significant change	Death	946	Yes	374	1a	0	0	1.6	Normal	Normal	Normal	Normal	Normal	Normal	1.10	A	well
1227	male	53	no significant change	high-expression	Death	143	Yes	84	4	1	0	15	Normal	>1ULN	Normal	Normal	Normal	>1ULN	1.10	A	moderately or poorly
1242	male	57	high-expression	no significant change	Death	1118	Yes	105	3	0	0	7.5	Normal	Normal	Normal	Normal	>1ULN	>1ULN	1.00	A	moderately or poorly
1246	male	50	high-expression	high-expression	Death	376	Yes	348	3	0	0	10	>1ULN	Normal	Normal	Normal	>1ULN	>3ULN	1.15	A	moderately or poorly
1257	male	46	high-expression	low-expression	Death	140	Yes	130	4	0	0	9	Normal	Normal	Normal	Normal	>1ULN	>3ULN	1.00	A	moderately or poorly
1260	male	70	no significant change	no significant change	Death	593	Yes	386	2	0	0	3.2	Normal	Normal	Normal	Normal	Normal	>1ULN	1.10	A	moderately or poorly
1276	male	62	no significant change	low-expression	Death	583	Yes	386	3	0	0	13	Normal	>1ULN	>1ULN	Normal	Normal	>3ULN	0.83	A	moderately or poorly
1277	female	49	high-expression	high-expression	Alive	2587	No	2587	1b	0	0	2	Normal	Normal	Normal	Normal	>1ULN	>1ULN	1.20	A	well
1295	male	45	no significant change	high-expression	Death	324	Yes	219	3	0	0	6	>1ULN	>1ULN	Normal	Normal	Normal	>1ULN	1.10	A	moderately or poorly
1297	male	60	no significant change	low-expression	Alive	1945	No	1945	1b	0	0	3.5	Normal	Normal	Normal	Normal	Normal	Normal	1.00	A	moderately or poorly
1315	male	68	high-expression	high-expression	Death	445	Yes	220	1a	0	0	2.5	>1ULN	>1ULN	>1ULN	Normal	>1ULN	>3ULN	1.10	A	moderately or poorly
1322	male	60	low-expression	no significant change	Alive	2527	Yes	1117	2	0	0	4.4	Normal	Normal	Normal	Normal	>1ULN	Normal	1.18	A	moderately or poorly
1343	male	28	low-expression	no significant change	Alive	2474	No	2474	1a	0	0	20	Normal	Normal	Normal	Normal	Normal	Normal	1.09	A	well
1365	male	59	high-expression	high-expression	Death	351	Yes	169	3	0	0	7	Normal	Normal	Normal	Normal	Normal	Normal	0.95	A	moderately or poorly
1402	male	62	low-expression	low-expression	Alive	2388	No	2388	2	0	0	4	Normal	Normal	>1ULN	Normal	Normal	Normal	1.02	A	well
1417	male	49	high-expression	high-expression	Alive	2356	No	2356	1b	0	0	4	>1ULN	>1ULN	Normal	Normal	Normal	>1ULN	1.11	A	moderately or poorly
1429	male	54	no significant change	high-expression	Alive	3359	Yes	2016	1b	0	0	4	Normal	Normal	Normal	Normal	Normal	Normal	0.98	A	well
1439	female	50	low-expression	no significant change	Death	305	Yes	277	3	0	0	7	Normal	Normal	Normal	Normal	Normal	Normal	1.04	A	moderately or poorly
1458	female	67	high-expression	no significant change	Death	1122	Yes	1122	3	0	0	10	Normal	Normal	Normal	Normal	Normal	Normal	0.92	A	moderately or poorly
1468	male	56	no significant change	low-expression	Alive	2255	Yes	761	1b	0	0	2.5	>1ULN	Normal	Normal	Normal	Normal	>1ULN	1.15	A	well
1493	male	62	low-expression	low-expression	Alive	2224	Yes	555	3	0	0	8	Normal	Normal	Normal	Normal	Normal	Normal	0.98	A	well
1501	male	62	low-expression	low-expression	Alive	2215	No	2215	3	0	0	12	>1ULN	>1ULN	Normal	Normal	>3ULN	>1ULN	1.08	A	well
1502	female	65	high-expression	no significant change	Alive	2219	Yes	1676	3	0	0	7	>1ULN	>1ULN	Normal	Normal	>1ULN	>1ULN	0.95	A	moderately or poorly
1504	male	66	low-expression	no significant change	Death	279	Yes	86	3	0	0	5.6	>1ULN	>1ULN	Normal	Normal	>1ULN	>3ULN	1.05	A	moderately or poorly
1512	male	35	low-expression	low-expression	Alive	2208	No	2208	1b	0	0	3.5	Normal	Normal	Normal	Normal	Normal	Normal	0.96	A	well
1529	female	44	low-expression	high-expression	Alive	2192	No	2192	2	0	0	5.5	Normal	Normal	>1ULN	Normal	>1ULN	Normal	0.92	A	well
1531	male	56	high-expression	high-expression	Death	869	Yes	324	1b	0	0	3.5	Normal	Normal	Normal	Normal	Normal	Normal	1.08	A	moderately or poorly
1532	female	69	low-expression	no significant change	Alive	1694	No	1694	1b	0	0	6	Normal	Normal	Normal	Normal	Normal	Normal	0.98	A	moderately or poorly
1655	male	55	high-expression	high-expression	Death	573	Yes	125	4	x	0	5	>1ULN	Normal	Normal	Normal	Normal	>1ULN	0.96	A	moderately or poorly
1660	male	57	no significant change	low-expression	Death	187	Yes	98	3	0	0	6	>1ULN	>1ULN	Normal	Normal	Normal	>1ULN	0.98	A	moderately or poorly
1663	male	63	no significant change	high-expression	Alive	2037	Yes	560	2	0	0	5	Normal	Normal	Normal	Normal	Normal	Normal	0.97	A	moderately or poorly
1670	male	46	no significant change	low-expression	Death	1295	Yes	1024	2	0	0	1.3	Normal	Normal	Normal	Normal	Normal	>1ULN	1.14	A	moderately or poorly
1676	female	37	no significant change	no significant change	Death	626	Yes	198	4	0	0	6.6	Normal	Normal	Normal	Normal	Normal	Normal	1.00	A	moderately or poorly
1677	male	63	high-expression	high-expression	Alive	2022	Yes	1055	3	0	0	9.5	>3ULN	>3ULN	Normal	Normal	Normal	>1ULN	0.98	A	moderately or poorly
1706	male	60	low-expression	low-expression	Alive	1968	No	1968	1b	0	0	3	Normal	Normal	Normal	Normal	Normal	Normal	1.07	A	moderately or poorly
1718	male	75	no significant change	no significant change	Death	1611	Yes	876	1b	0	0	2.7	Normal	Normal	Normal	Normal	Normal	>1ULN	0.91	A	moderately or poorly
1748	female	58	no significant change	high-expression	Alive	2906	Yes	2449	2	0	0	6.5	Normal	Normal	Normal	Normal	Normal	Normal	1.30	A	moderately or poorly

Supplementary Table S7 Clinical data of 50 HCC patients in our study. OS,overall suvival;RFS, Recurrence-free survival;ALB, albumin;TBIL, total bilirubin;AST, aspartate aminotransferase; ALT, alanine aminotransferase; GGT, gamma glutamyl transferase; ALP, alkaline phosphatase; INR, international normalized ratio; ULN, upper limitof normal value;Tumor size was defined as the sum of the longest diameters of target lesions. TNM staging was classified using AJCC 8th edition.

Name	Forward(5'-3')	Reverse(5'-3')
LUCAT1	GGATAAACAGAGGCAACCCGA	GACTGCAAGAGCTTGAAGGCT
AC015908.3	CTGATACCTCCACAGTTATTCCG	AGGCTAAGGAAATAGGCTCAGG
AL031985.3	AGACCCACTGATGAATGTGTGC	CTTGAGCCAAACGAAACCTAAC
GAPDH	TGTCAAGCTCATTTCCTGGTATG	TCTCTCTTCCTCTTGTGCTCTTG

Supplementary Table S8 Real-time quantitative PCR primer sequences used in this study.
